# Potent type-specific de novo antibodies complement broadly reactive imprinted antibodies in immune responses to SARS-CoV-2 variants

**DOI:** 10.1038/s41590-026-02613-4

**Published:** 2026-08-14

**Authors:** Timothy S. Johnston, Shuk Hang Li, Mark M. Painter, Divya Swaminathan, Reilly K. Atkinson, Bernadeta Dadonaite, Shuishu Wang, Naomi R. Douek, Lucas Kampman, Robin Schlesinger, Rachel Kazmierski, Bob C. Lin, Leonid A. Serebryannyy, Haijuan Du, Amy R. Henry, Sarah C. Smith, Farida Laboune, I-Ting Teng, Lingshu Wang, Nicole A. Doria-Rose, Chaim A. Schramm, Theodore C. Pierson, Tongqing Zhou, Jesse D. Bloom, E. John Wherry, Scott E. Hensley, Daniel C. Douek

**Affiliations:** 1 Vaccine Research Center, National Institute of Allergy and Infectious Diseases, National Institutes of Health, Bethesda, MD USA; 2 Department of Microbiology, University of Pennsylvania Perelman School of Medicine, Philadelphia, PA USA; 3 Institute for Immunology and Immune Health, University of Pennsylvania Perelman School of Medicine, Philadelphia, PA USA; 4 Department of Systems Pharmacology and Translational Therapeutics, University of Pennsylvania Perelman School of Medicine, Philadelphia, PA USA; 5 Basic Sciences Division and Computational Biology Program, Fred Hutchinson Cancer Center, Seattle, WA USA; 6 Howard Hughes Medical Institute, Seattle, WA USA

**Keywords:** Antibodies, RNA vaccines, SARS-CoV-2, Immunological memory

## Abstract

For rapidly mutating viruses such as influenza viruses and severe acute respiratory syndrome coronavirus 2 (SARS-CoV-2), immune memory recalled by antigenically drifted variants primarily comprises antibodies that cross-react to the priming strain rather than de novo elicited responses, a phenomenon termed original antigenic sin or immune imprinting. The composition and functionality of de novo responses elicited by variant exposures remain unclear. Here we isolated and characterized hundreds of recall and de novo neutralizing monoclonal antibodies after sequential exposures to SARS-CoV-2 variants in ancestral-imprinted humans. De novo variant type-specific antibodies used different V(D)J genes that were closer to germline sequence, potently neutralized future variants and targeted distinct receptor binding domain epitopes compared to ancestral cross-reactive (recall) antibodies. Nevertheless, neutralizing responses to the updated 2024–2025 booster were predominantly ancestral cross-reactive. These results reveal the distinct contributions of recall and de novo antibodies to a balanced immune response and underscore the benefit of updated booster vaccines, which augment both subsets.

## Abstract (web summary)

Updated SARS-CoV-2 boosters broaden humoral immunity by recalling cross-reactive antibodies and eliciting new less cross-reactive Omicron type-specific antibodies that target distinct RBD epitopes and more potently neutralize recent variants.

## Main

The emergence of severe acute respiratory syndrome coronavirus 2 (SARS-CoV-2) in 2019 was followed by rapid development of vaccines based on ancestral spike glycoprotein sequences (collectively referred to as WA1)^1–3^. These vaccines demonstrated high early efficacy, preventing an estimated 19.8 million deaths in their first year of use^4,5^. Following the initial outbreak, antigenic drift and larger saltatory evolutionary events led to accumulation of mutations in the spike glycoprotein, resulting in a rapid increase in antigenic diversity and emergence of variants that escaped vaccine- and infection-elicited antibodies^6–13^. As is the case with influenza virus, immune escape variants continue to pose a threat to public health, resulting in seasonal increases in infections and hospitalizations. In response to major shifts in antigenic distance, five booster vaccine updates have been issued with the goal of increasing neutralizing antibody (nAb) titers against circulating Omicron subvariants: a WA1-BA.5 bivalent formulation in 2022, XBB.1.5 in 2023, KP.2 in 2024 and LP.8.1 in 2025 (refs. ^14–20^). Consequently, populations have recently experienced sequential exposures to an increasingly different antigen in the context of primary ancestral immunity, resulting in a complex immune landscape akin to what has been observed with influenza viruses.

Sequential exposures to progressively mutating antigens have been found to result in important epidemiological and immunological consequences. The recall of antibodies elicited by primary responses after subsequent exposure to a different but antigenically related strain (termed ‘original antigenic sin’ or ‘immune imprinting’) has been studied at the epidemiological level and also at the individual level in terms of B cell responses^21–24^. Several examples from epidemiological influenza research suggest that initial subtype exposure may reduce the risk of infection to certain subtypes later in life^25–28^, which is hypothesized to be driven by antibody cross-reactivity to related but unencountered antigens^29,30^. Although the underlying mechanisms are unclear, influenza virus exposure history is also suggested to contribute to decreased seasonal vaccine efficacy and increased disease burden during outbreaks of novel influenza viruses^31–35^. Thus, imprinting may be beneficial or detrimental at the epidemiological level depending on the circumstances.

Recent studies investigating human immune responses after exposure to SARS-CoV-2 variants in preimmune individuals have focused on the effects of imprinting on B cell responses. Several studies have found evidence of persistent recall of ancestral cross-reactive B cells and antibodies, rarely observing variant type-specific antibodies despite multiple variant exposures^36–42^. However, recent work in mouse models and humans who have been infected multiple times after being initially exposed to inactivated virus-based vaccines has shown that repeated exposures to novel variants can elicit type-specific antibodies, suggesting that the nature and consequences of immune imprinting may differ among vaccine platforms^43,44^. Yet, the mechanistic factors that determine the balance between memory recall and de novo responses after sequential antigenic exposures are not well understood, including the conditions under which reliance on immune memory may be overcome and the potential benefit to eliciting de novo responses.

To understand the provenance and functionality of de novo SARS-CoV-2 responses compared to imprinted recall responses, we isolated and characterized 1,013 variant receptor binding domain (RBD)-reactive monoclonal antibodies (mAbs) from individuals before and after SARS-CoV-2 variant exposures. We compared sequence, potency and epitope specificity among WA1 cross-reactive and Omicron type-specific nAbs. We found newly elicited type-specific antibodies to be distinct in their immunoglobulin gene usage and somatic hypermutation (SHM), functionality and epitope specificity. Furthermore, even though Omicron type-specific antibodies could be elicited by updated booster vaccination, neutralizing responses were nevertheless dominated by recall of cross-reactive memory.

## Results

### Variant-specific mAb isolation

We previously assessed serum antibodies to SARS-CoV-2 in a cohort of individuals that received more than three WA1 mRNA lipid nanoparticle (mRNA-LNP) vaccines and a subsequent Omicron XBB subvariant exposure by infection or mRNA-LNP vaccination. Using a serum absorption assay, we depleted spike-reactive antibodies to WA1 from sera and measured the binding and neutralization titers of the remaining fraction. Although WA1 absorption depleted all nAbs in most individuals, we identified 5 of 22 individuals that had a detectable titer of nAbs to XBB.1.5 that were not depleted by WA1 spike absorption, providing evidence of an Omicron type-specific response (herein referred to as W^−^O^+^)^40^.

**Fig. 1:**
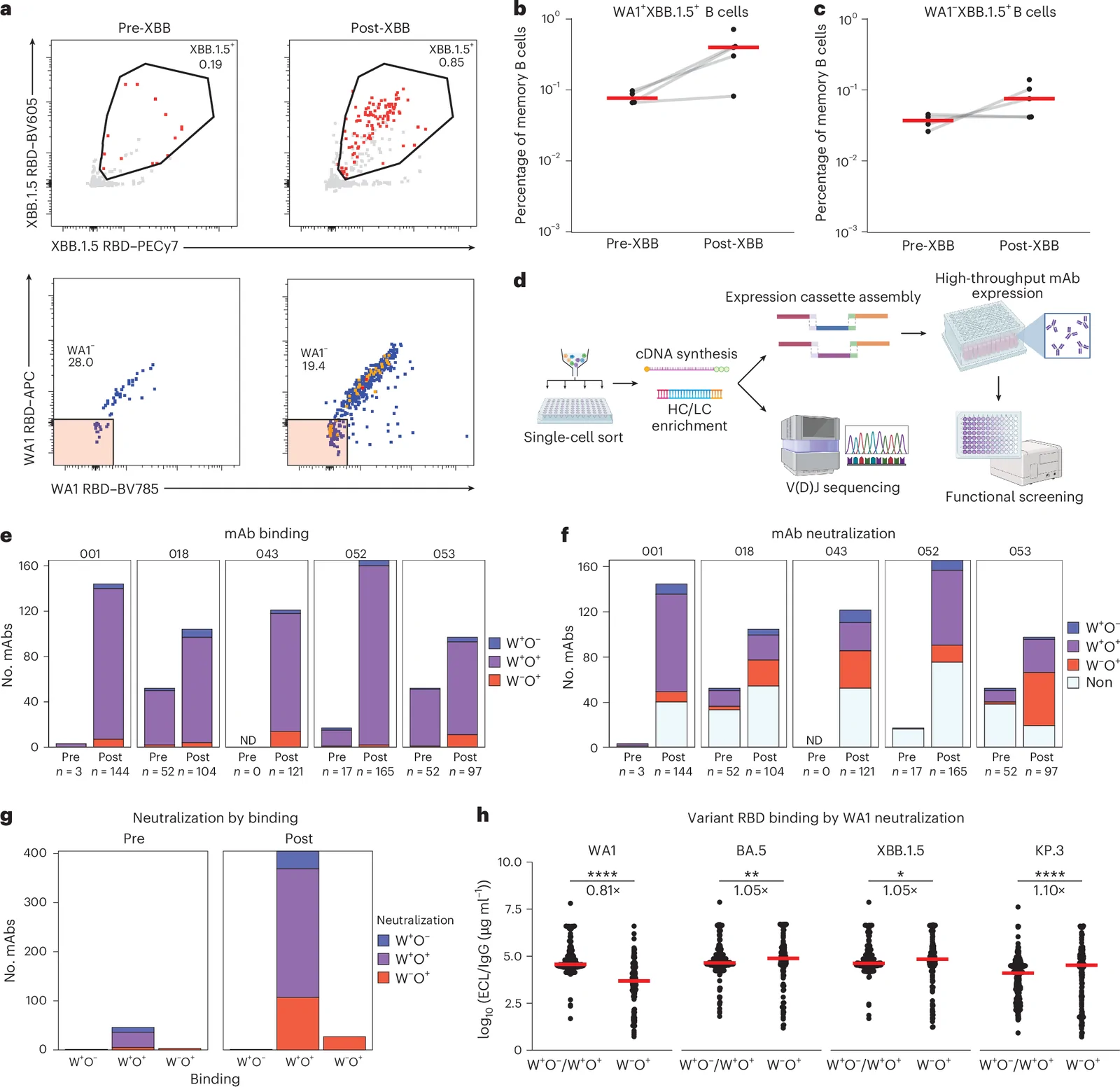
XBB exposure elicits type-specific antibodies and B cells. **a**, RBD probe binding of memory B cells sorted from donors with type-specific sera. Red cells indicate back gating of WA1 RBD^−^ cells below. **b**,**c**, Frequencies of WA1^+^XBB.1.5^+^ (**b**) and WA1^−^XBB.1.5^+^ (**c**) memory B cells before and after XBB exposure (*n* = 4 individuals pre-XBB and *n* = 5 individuals post-XBB). Light gray lines connect donors. Crossbars represent medians. **d**, Schematic of the RATP-Ig workflow used to isolate mAb supernatants and V(D)J sequences; HC, heavy chain; LC, light chain. **e**,**f**, Variants bound (**e**) and neutralized (**f**) by mAb supernatants from each donor before and after XBB exposure. The total numbers of mAbs isolated at each time point are reported below; Non, nonneutralizing; ND, not detected. **g**, Variant neutralization by binding status before and after XBB exposure. **h**, IgG normalized binding values to variant RBDs for W^+^O^−^/W^+^O^+^ (*n* = 344) and W^−^O^+^ (*n* = 160) nAbs (WA1 *P* = 8.24 × 10^−39^; BA.5 *P* = 1.49 × 10^−3^; XBB.1.5 *P* = 1.01 × 10^−2^; KP.3 *P* = 7.62 × 10^−6^); ECL, enhanced chemiluminescence. Crossbars represent the median. Data were analyzed by Wilcoxon rank-sum test with a Benjamini–Hochberg correction for multiple testing; *P < 0.05; **P < 0.01; ****P < 0.0001. All tests performed were two sided. Diagram in **d** created in BioRender; Fabozzi, G. https://biorender.com/ezh01v0 (2026). Source data

**Extended Data Fig. 1:**
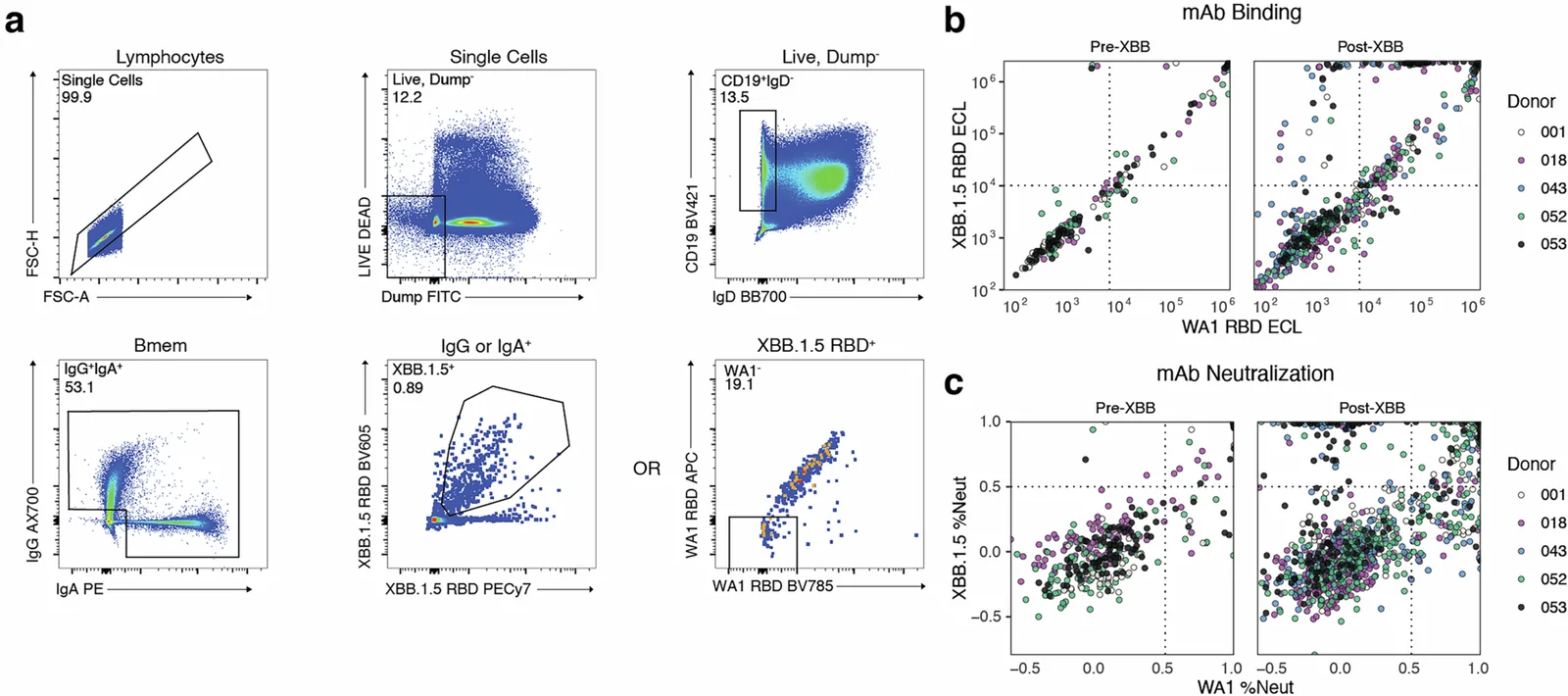
Sorting strategy and mAb screening. **a**) Sorting strategy for RBD probe^+^ memory B cells. **b**) MSD binding of mAb supernatants. Each dot represents one supernatant, colored by donor. Dotted line represents positive cutoff. **c**) One-point pseudovirus neutralization of mAb supernatants. Each dot represents one supernatant, colored by donor. Dotted line represents positive cutoff. Source data

To validate the existence of W^−^O^+^ antibodies and compare them to WA1 cross-reactive (W^+^O^+^) antibodies, we used fluorophore-conjugated RBD tetramers to sort class-switched B cells that bound XBB.1.5 RBD from these five identified individuals before and after XBB exposure. To further enrich for XBB type-specific B cells, we also sorted XBB.1.5^+^ B cells that did not bind WA1 RBD tetramer (Fig. 1a and Extended Data Fig. 1a). Frequencies of WA1^+^XBB.1.5^+^ cross-reactive and WA1^−^XBB.1.5^+^ Omicron type-specific memory B cells increased after XBB exposure (Fig. 1b,c).

To express mAbs from sorted cells, we performed rapid assembly, transfection and production of immunoglobulins (RATP-Ig^45^; Fig. 1d). We expressed a total of 1,359 mAbs, screened supernatants for binding to WA1, BA.5, XBB.1.5 and KP.3 variant RBDs and measured their ability to neutralize WA1, BA.5, XBB.1.5 and KP.3 pseudoviruses (Extended Data Fig. 1b,c). We recovered paired heavy and light chain sequences for 1,013 mAbs. mAbs were binned into W^+^O^−^, W^+^O^+^ or W^−^O^+^ reactivity groups based on their ability to bind and neutralize WA1 and/or any Omicron variants (BA.5, XBB.1.5 or KP.3). Most isolated mAbs demonstrated a high binding signal to all RBDs tested, although we found an increase in the proportion of mAbs that only bound Omicron variants after XBB.1.5 boost (Fig. 1e). In every donor, we observed an increase in the number and frequency of neutralizing mAbs, particularly those that only neutralized Omicron variants (W^−^O^+^; Fig. 1f).

When comparing binding versus neutralizing capacity of mAbs, we found that although many mAbs were found to be cross-reactive binders of both WA1 and Omicron variants (W^+^O^+^), there were also type-specific neutralizers of either WA1 or Omicron variants (W^+^O^−^ or W^−^O^+^; Fig. 1g). This finding revealed an important disparity between the ability of antibodies to bind and neutralize variants. Although nAbs reactive to WA1 demonstrated significantly higher median binding affinity to WA1 RBD, antibodies specific to Omicron demonstrated a significantly higher median binding affinity to BA.5, XBB.1.5 and KP.3 RBDs (Fig. 1h). These results suggest that type-specific antibody responses can be elicited in WA1 mRNA-imprinted individuals following variant exposure.

### Comparison of type-specific and cross-reactive neutralizing mAbs

**Fig. 2:**
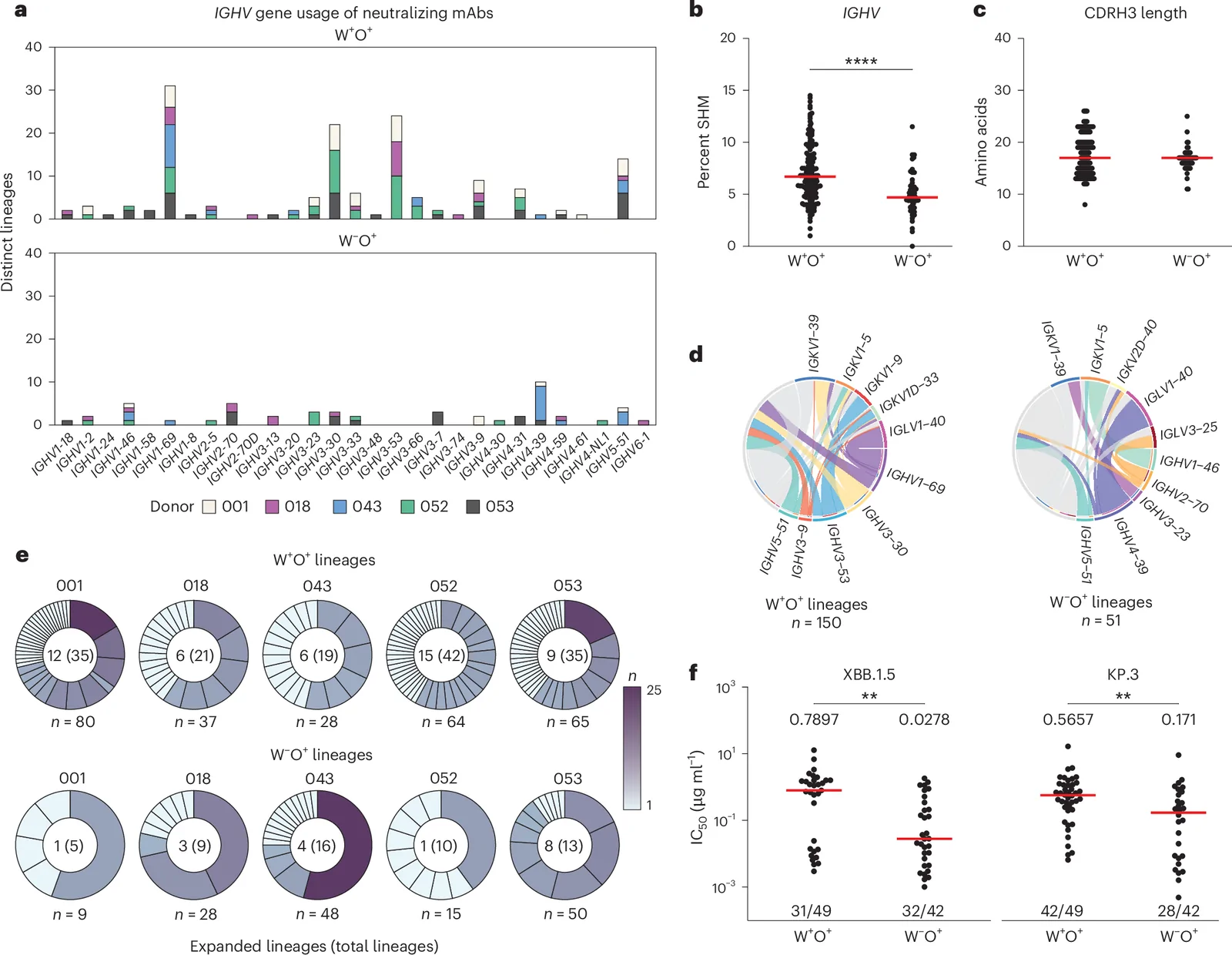
Comparison of de novo and imprinted antibodies. **a**, Number of mAb lineages expressing each *IGHV* gene in W^+^O^+^ and W^−^O^+^ nAbs, colored by donor. **b**, Mean *IGHV* gene SHM percentage compared to germline sequence in W^+^O^+^ (*n* = 150) and W^−^O^+^ (*n* = 51) nAb lineages (*P* = 5.6 × 10^−7^). **c**, CDRH3 amino acid lengths of W^+^O^+^ (*n* = 150) and W^−^O^+^ nAb lineages (*n* = 51). **d**, Circos plots of heavy chain–light chain pairings in W^+^O^+^ and W^−^O^+^ nAb lineages. The top five heavy and light chain gene usages are in color, and all others are in gray. Threads connect paired heavy and light chain usages. For heavy chain sections, the inner colored segment represents light chain pairing. **e**, Ring plots of clonal expansion in each donor for W^+^O^+^ and W^−^O^+^ antibodies. Expanded lineages are purple. For inner numbers, the first number reports expanded lineages, and the total lineages recovered are in parentheses. Total numbers of nAbs are reported below each ring. **f**, XBB.1.5 and KP.3 pseudovirus IC_50_ values of W^+^O^+^ (XBB.1.5 *n* = 31; KP.3 *n* = 42) and W^−^O^+^ (XBB.1.5 *n* = 42; KP.3 *n* = 28) nAbs (XBB.1.5 *P* = 0.0024; KP.3 *P* = 0.005). Median IC_50_ values are reported above. IC_50_ values were interpolated from duplicate measurements. Crossbars represent median values. For **b**, **c**, **f** and **g**, data were analyzed by Wilcoxon rank-sum test; **P < 0.01; ****P < 0.0001. All tests performed were two sided. Source data

**Extended Data Fig. 2:**
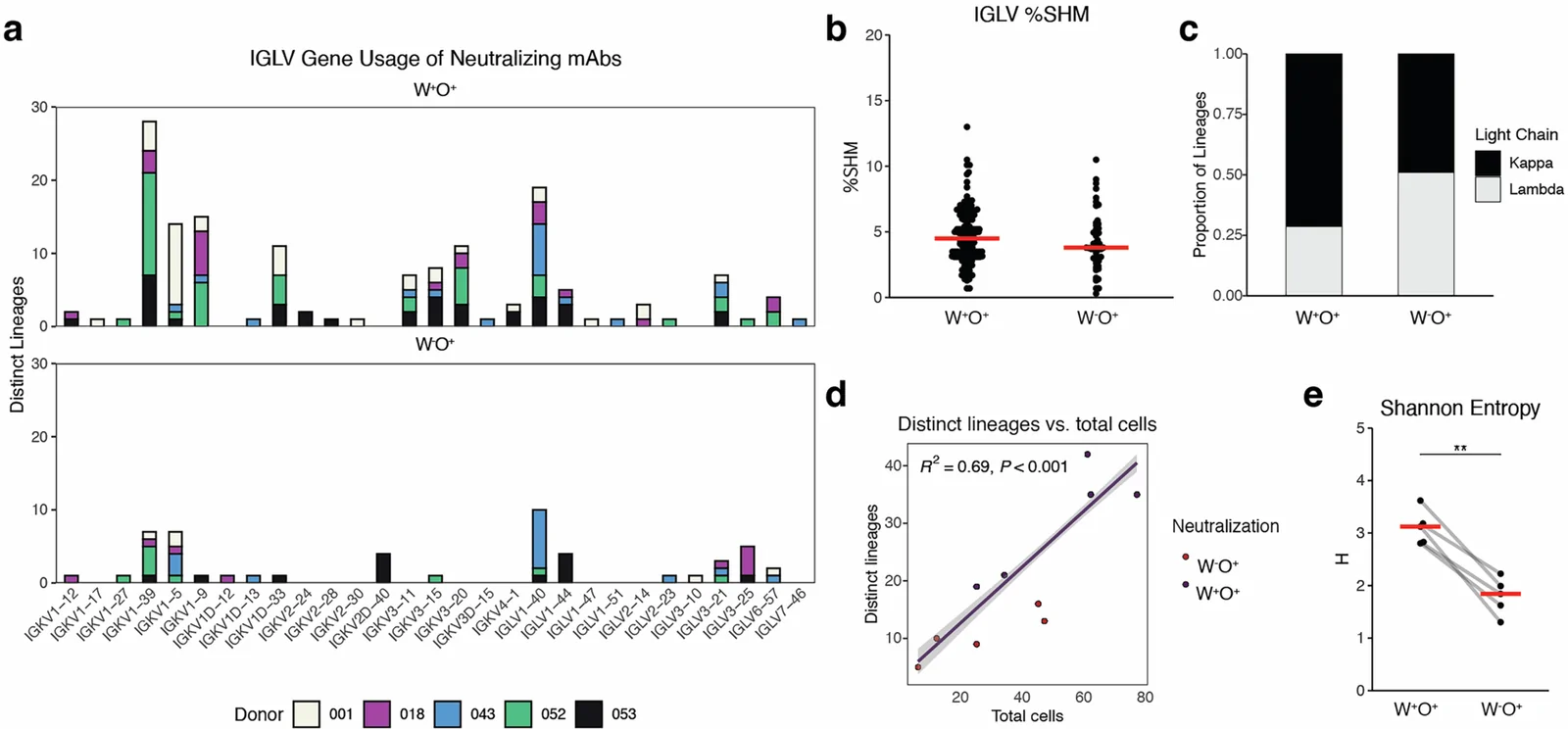
Additional mAb sequence analysis. **a**) Light chain gene usage for W^+^O^+^ (above) and W^−^O^+^ (below) distinct neutralizing mAbs lineages. Number of lineages is colored by donor. **b**) Light chain somatic hypermutation of W^+^O^+^ and W^−^O^+^ lineages. Crossbars represent median. **c**) Proportion of kappa vs. lambda light chains being used in W^+^O^+^ and W^−^O^+^ lineages. **d**) Relationship between number of distinct lineages identified versus total cells recovered. Each dot represents one donor’s W^+^O^+^ or W^−^O^+^ lineages. Linear regression line, R^2^ value, and significance are presented. Shaded band represents 95% CI around the fitted regression line. e) Shannon entropy of W^+^O^+^ and W^−^O^+^ lineages for each donor (*n* = 5), connected by lines (*P* = 0.008). Crossbars represent median. Wilcoxon rank sum test, **p < 0.01. All tests performed were two-sided. Source data

We next sought to compare the immunoglobulin variable gene expression and SHM of the mAbs that neutralized both the WA1 and Omicron variants (W^+^O^+^) or only neutralized Omicron variants but not WA1 (W^−^O^+^). Here, we focused on the 424 nAbs comprising 201 distinct lineages. W^+^O^+^ nAb lineages were dominated by genes including *IGHV1-69*, *IGHV3-30* and *IGHV3-53* frequently used across multiple donors (Fig. 2a). W^−^O^+^ nAb lineages had little *IGHV* gene overlap with the cross-reactive antibodies and usage of a particular *IGHV* gene restricted to one or two donors in most cases (Fig. 2a). W^−^O^+^ nAb lineages demonstrated significantly lower levels of heavy chain variable region SHM with a median of 4.4% compared to 6.5% in W^+^O^+^ nAbs. Although both groups had median CDRH3 lengths of 17 amino acids, they displayed different distributions where cross-reactive antibodies skewed short or long in a bimodal distribution, while type-specific antibodies showed a unimodal distribution (Fig. 2b,c). *IGLV* gene usage differences were less marked; however, we noted a similar trend in lower SHM and a large increase in frequency of immunoglobulin λ-chain usage in W^−^O^+^ lineages (Extended Data Fig. 2a–c). Together, these *IGHV* and *IGLV* genes formed distinct pairs, displaying almost no overlap between neutralizing specificities, with only 2% of lineages identified having antibodies with both W^+^O^+^ and W^−^O^+^ neutralization (Fig. 2d). Although expanded lineages appeared to occur more frequently among cross-reactive nAbs, which is likely due to total cells recovered, the type-specific nAb repertoire was significantly less diverse as calculated by Shannon entropy, suggesting a more focused response (Fig. 2e and Extended Data Fig. 2d,e).

We next compared the relative ability of the type-specific nAbs and WA1 cross-reactive nAbs to neutralize future variants, measuring the half-maximum inhibitory concentration (IC_50_) values for a subset of nAbs representing distinct lineages from each group to XBB.1.5 and KP.3 pseudoviruses. W^−^O^+^ nAbs were significantly more potent neutralizers of both XBB.1.5 and KP.3 pseudoviruses, with median IC_50_ differences of 28.4- and 3.3-fold, respectively (Fig. 2f). However, although type-specific nAbs were on average more potent, we also observed a subset of highly potent W^+^O^+^ nAbs that was mainly composed of *IGHV3-53* lineages but also included *IGHV1-58*, *IGHV1-69*, *IGHV3-30* and *IGHV5-51*.

Together, these results suggest that after Omicron variant exposure, novel Omicron type-specific B cell lineages with low levels of SHM arise that potently neutralize Omicron variants, and individuals may also harbor highly mutated WA1 cross-reactive lineages of equal potency. Because the type-specific antibodies to Omicron we identified had significantly lower affinity for the WA1 variant and fewer SHMs and used different *IGHV* and *IGLV* genes, these antibodies are likely of de novo origin rather than imprinted recall antibodies being redirected toward Omicron variants through sequential exposure.

### Comparison of mAb epitopes

**Fig. 3:**
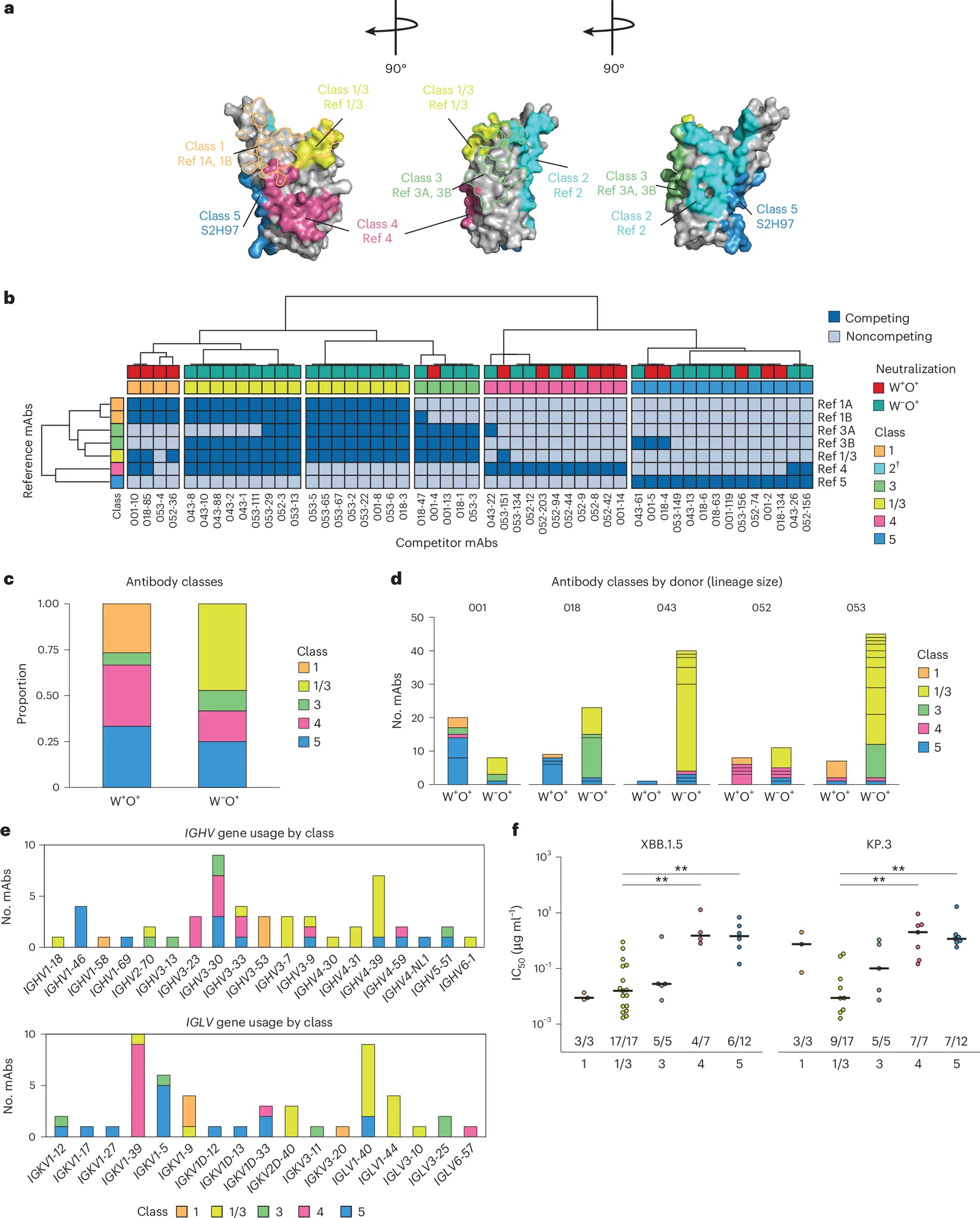
Epitope mapping of type-specific mAbs. **a**, RBD structure (Protein Data Bank (PDB): 7M7W)^48^ colored with different reference antibody footprints. **b**, Map of epitope binning assay results. Rows represent surface reference mAbs, and columns represent flowover competitor mAbs (*n* = 51). The color of the inner squares denotes blocking or non-blocking interactions. Ward’s hierarchical method was used for mAb clustering. Outer squares annotate binding class (rows and columns) or neutralization ability (columns). Data were compiled from two experiments performed on the same antibody surface. **c**, Summary of antibody binding classes of W^+^O^+^ and W^−^O^+^ mAbs. **d**, Antibody classes separated by donor and accounting for lineage sizes. Within each column, each segment represents one lineage. **e**, *IGHV* and *IGLV* gene usage of mAbs of different binding classes. **f**, XBB.1.5 and KP.3 pseudovirus IC_50_ values of different binding classes of nAbs (XBB.1.5 *P* = 0.0033; KP.3 *P* = 0.0024). The fraction of nAbs in each class that display variant neutralization is shown at the bottom (data are from W^+^O^+^ and W^−^O^+^ nAbs combined). Crossbars represent the median. In **f**, data were analyzed by Wilcoxon rank-sum test with a Benjamini–Hochberg correction for multiple testing; **P < 0.01. All tests performed were two sided. The dagger (†) indicates that class 2 reference mAb data were excluded due to data inconsistency and otherwise clear epitope clustering. Source data

The evidence suggesting that antibodies specific to Omicron are generated de novo raises the hypothesis that they target novel RBD epitopes on Omicron variants that are not present on WA1 RBD and are potentially physically distinct from primary WA1 epitopes. To classify the epitope targets of our mAbs, we performed competitive binding assays on type-specific mAbs and potent cross-reactive mAbs (termed ‘competitor mAbs’ in the assay) using a reference panel of antibodies (‘reference mAbs’) structurally characterized by cryo-electron microscopy that cover RBD epitopes defined according to the Barnes classification^46–48^ (Fig. 3a). Reference and competitor mAbs competed for binding to XBB.1.5 RBD, and binary outcomes were used to cluster mAbs based on reactivity patterns (Fig. 3b,c). W^+^O^+^ mAbs were enriched for class 1, 4 and 5 binding antibodies, whereas W^−^O^+^ mAbs were enriched for class 1/3 binding antibodies (Fig. 3b,c). After accounting for number of antibodies sequenced within each lineage, class 1, 4 and 5 mAbs predominated among most W^+^O^+^ mAbs, and class 1/3 or 3 predominated among W^−^O^+^ mAbs, although this differed by donor (Fig. 3d). Notably, no class 1/3 binders were found among the W^+^O^+^ nAbs, and no class 1 binders were found among the W^−^O^+^ nAbs, revealing distinct RBD epitope targeting between these two neutralizing specificities.

We observed distinct patterns of *IGHV* and *IGLV* gene usage among the different classes of mAbs. For heavy chains, class 1 antibodies were predominantly *IGHV3-53*, class 1/3 was distributed across *IGHV3-7*, *IGHV4-31* and *IGHV4-39*, and class 4 and 5 showed no distinct patterns (Fig. 3e). For light chains, class 1 antibodies were predominantly *IGKV1-9*, and class 1/3 antibodies were distributed across *IGKV2D-40*, *IGLV1-40* and *IGLV1-44*. Class 4 antibodies almost exclusively used *IGKV1-39*. Class 3 and 5 antibodies did not show distinct usage patterns (Fig. 3e). These data suggest that RBD binding classes have characteristic genetic and functional traits.

Finally, to compare the neutralization ability of the different classes of mAbs identified, we compared IC_50_ values of mAbs in each class against XBB.1.5 and KP.3 pseudoviruses. Class 1 and 1/3 mAbs were the most potent XBB.1.5 neutralizers. We found class 1 mAbs to be susceptible to mutations in KP.3, dropping by almost 100-fold in potency (Fig. 3f). Although there was no drop in average potency for class 1/3 mAbs, 8 of 17 mAbs were unable to neutralize KP.3 altogether (Fig. 3f). The remaining classes, while less potent against XBB.1.5, had little to no drop in potency against KP.3 (Fig. 3f).

Collectively, these results suggest distinct epitope targeting between recall and de novo nAb lineages, where recalled B cells target conserved sites with lower potency and newly elicited B cells recognize mutated residues on Omicron RBDs not present on WA1 RBD with high potency, often retaining neutralization breadth beyond the eliciting variant.

### Fine-grain mapping of type-specific antibodies

**Fig. 4:**
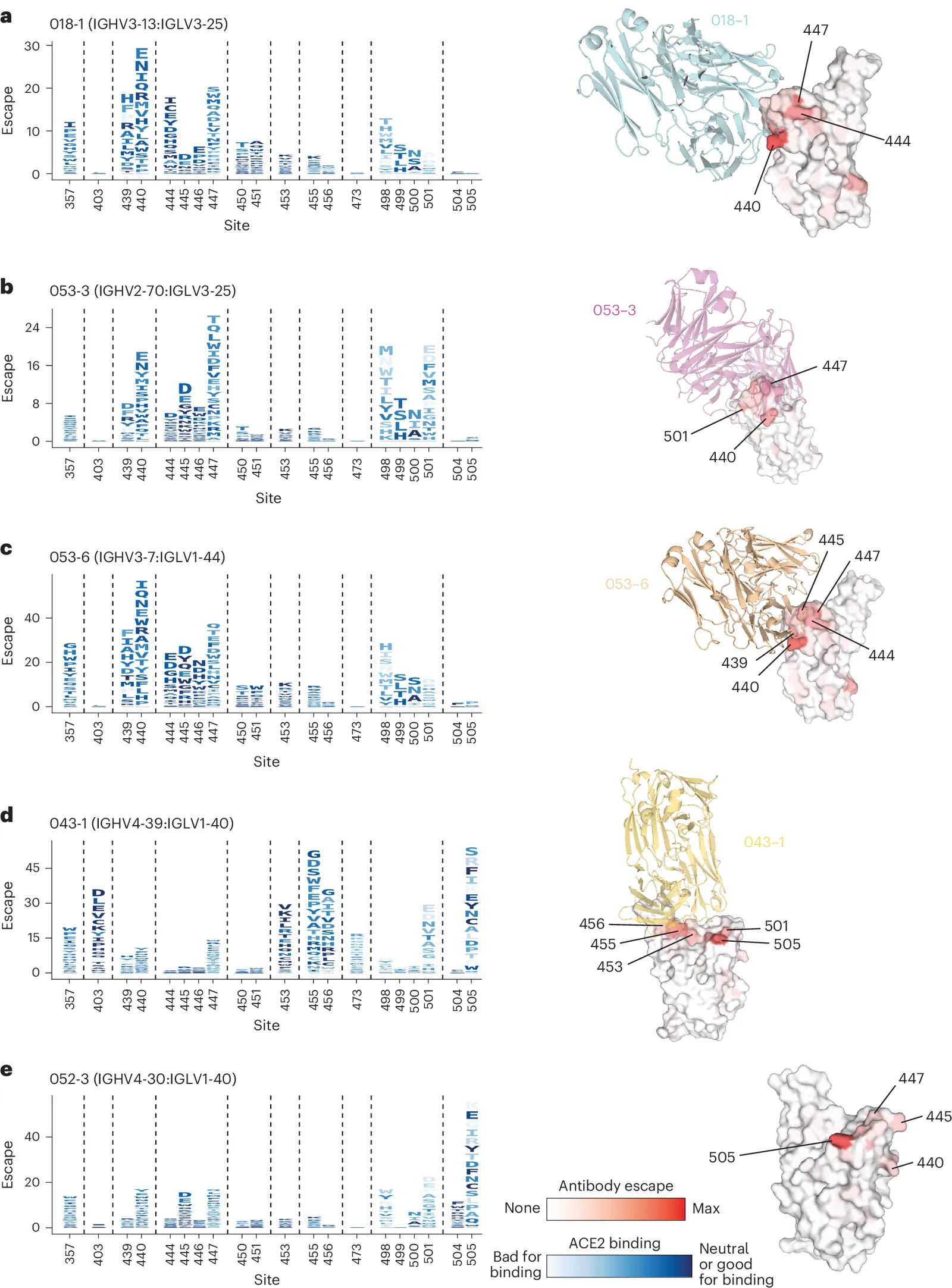
Fine-grain mapping of expanded type-specific lineages. **a**, Deep mutation scanning of 018-1 antibody escape. Logo plots on the left show escape caused by each measured mutation at key sites. The size of each letter indicates escape caused by a mutation; letters are colored by mutation effects on ACE2 receptor binding (as published in Dadonaite et al.^50^). The structure on the right shows the RBD (PDB: 8A94)^61^ with the 018-1 Fab, which was modeled using AlphaFold and negative-stain electron microscopy density maps. The surface of RBD is colored by the total site escape. **b**–**e**, Same as **a** but for 053-3 (**b**), 053-6 (**c**), 043-1 (**d**) and 052-3 (**e**), respectively. The Fab structure is not available for 052-3. For an interactive figure showing mutation-level escape across the full spike, see https://dms-vep.org/SARS-CoV-2_XBB.1.5_spike_DMS_NIH_Penn_mAbs/htmls/Antibody_escape_faceted.html.

**Extended Data Fig. 3:**
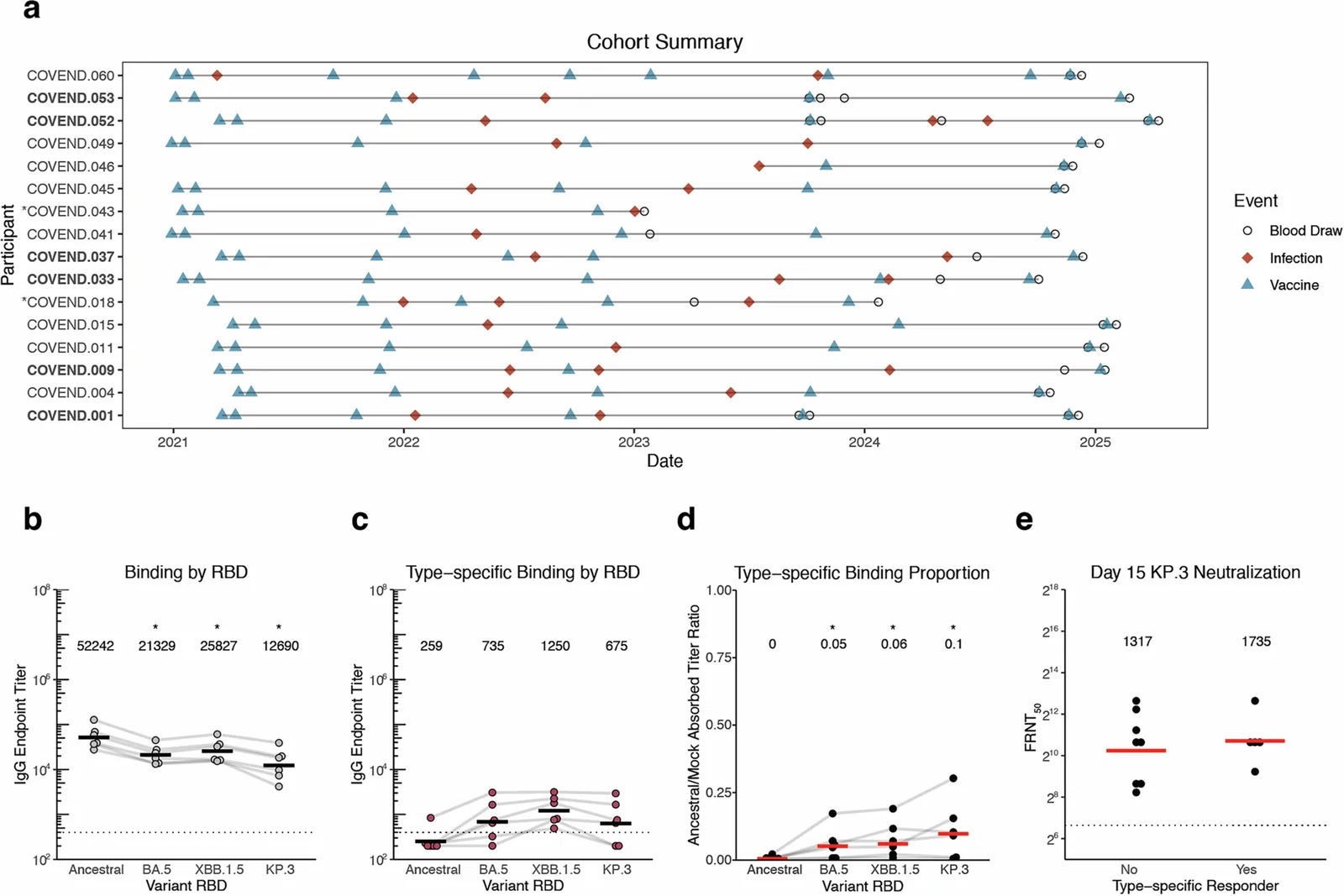
KP.2 vaccine cohort summary and additional serology. **a**) KP.2 booster cohort exposure histories and blood draw summary. Participants with type-specific serum responses are bolded. **b**) Of donors with type-specific responses (*n* = 6), Mock-absorbed serum RBD binding against ancestral, BA.5, XBB.1.5, and KP.3 RBDs by ELISA (BA.5 *P* = 0.31; XBB.1.5 *P* = 0.31; KP.3 *P* = 0.31). Crossbars represent geometric mean. Geometric mean titer values are displayed above points. **c**) Of donors with type-specific responses, ancestral-absorbed serum RBD binding against ancestral, BA.5, XBB.1.5, and KP.3 RBDs by ELISA. Geometric mean titer values are displayed above points. **d**) Values from b divided by values from c, or the proportion of the overall responses that is not absorbable by ancestral spike (BA.5 *P* = 0.31; XBB.1.5 *P* = 0.31; KP.3 *P* = 0.31). **e**) Day 15 post-boost KP.3 pseudovirus neutralization titer comparison of people with and without variant type-specific serum responses. Geometric mean titer values are displayed above points. For b, c, and e, red or black bars indicate geometric mean. For d, red bars indicate median. Wilcoxon signed-rank test with Benjamini-Hochberg correction for multiple testing. Statistical comparisons to first time point ancestral (**c**-**e**). *p < 0.05. Donors COVEND.018 and COVEND.043 provided samples for the XBB type-specific response cohort, but not the KP.2 booster cohort. All tests performed were two-sided. Source data

To perform fine-grain mapping of residues in RBD that affect antibody neutralization for representative class 1/3 and 3 W^−^O^+^ mAb lineages, we used previously described^49^ XBB.1.5 spike pseudovirus-based deep mutational scanning libraries (Fig. 4). This analysis showed that mAbs 018-1, 053-6 and 053-3 have similar neutralizing epitopes that are focused on RBD sites 439–440, 444–447 and 498–501 (Fig. 4a–c). Mutations at sites 403, 453, 455–456, 501 and 505 have strong effects on mAb 043-1 (Fig. 4d), while mAb 052-3 was primarily affected by mutations at sites 501 and 505 (Fig. 4e). Note that all antibodies are affected by mutations at sites such as 357 that are distal from the physical epitope but affect RBD up/down motion and hence sensitivity to neutralization by RBD-directed antibodies^50^. To further confirm binding profiles of these five mAbs, we performed negative-stain electron microscopy to image Fab complexed with XBB.1.5 spike protein (Fig. 4). Three-dimensional reconstructions were generated for all Fabs except 052-3, which dissociated the spike trimer. mAbs 018-1 and 053-3 were predicted to bind residues 440–449 and 495–503 in the same mode (Fig. 4a,b). Models for mAb 053-6 predicted similar residues as mAbs 018-1 and 053-3, including binding interactions with 438–451 and 496–501 (Fig. 4c). mAb 043-1 was predicted to bind residues 403, 455/456 and 505. In all cases, binding and neutralization escape predictions were harmonious and in agreement with our epitope binning experiments, defining critical epitopes for variant type-specific antibodies. Mutations at the critical escape residues (440, 498, 501 and 505) have been present in the RBD since early Omicron circulation before XBB. These observations suggest that these B cells may have been seeded by prior Omicron exposures and were subsequently recalled by XBB exposure, as all but one donor had one to two previous Omicron exposures through vaccination and/or infection (Extended Data Fig. 3a).

### Type-specific serum reactivity after KP.2 boost

**Fig. 5:**
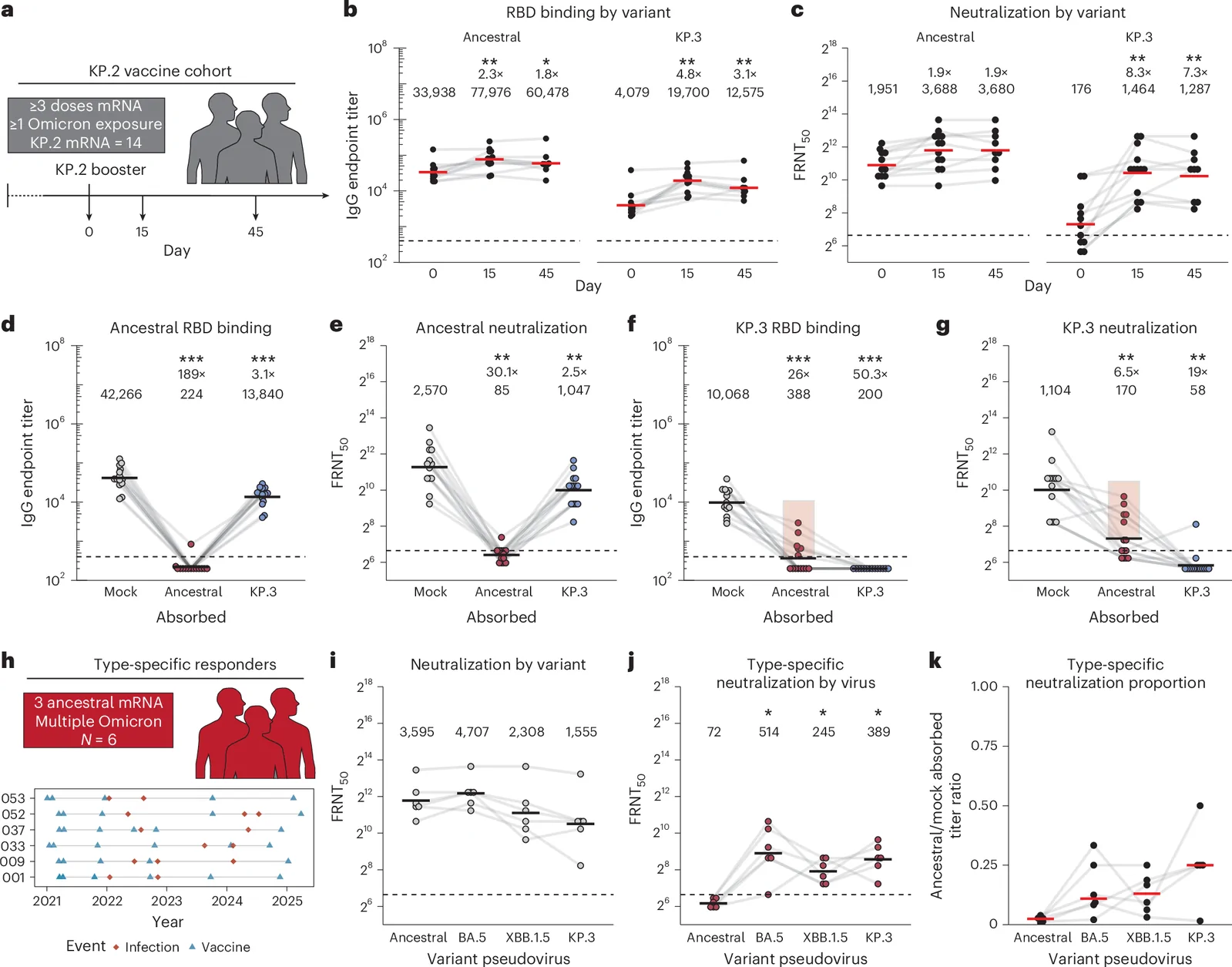
Antibody response to antigenically distant KP.2 boost. **a**, KP.2 cohort overview. **b**, Reciprocal end point titers of antigen-specific IgG to ancestral (day 15 *P* = 0.004; day 45 *P* = 0.025) and KP.3 RBDs (day 15 *P* = 0.0012; day 45 *P* = 0.004) measured by enzyme-linked immunosorbent assay (ELISA). **c**, Focus reduction neutralizing titers of ancestral and KP.3 (day 15 *P* = 0.0018; day 45 *P* = 0.003) pseudoviruses; FRNT_50_, half-maximum focus reduction neutralization titer. **d**, Ancestral RBD binding of absorbed sera from day 15 to day 45 post-KP.2 boost (ancestral *P* = 2.44 × 10^−4^; KP.3 *P* = 2.44 × 10^−4^). **e**, Ancestral pseudovirus neutralization of absorbed sera from day 15 to day 45 post-KP.2 boost (ancestral *P* = 0.002; KP.3 *P* = 0.002). **f**, KP.3 RBD binding of absorbed sera from day 15 to day 45 post-KP.2 boost (ancestral *P* = 2.44 × 10^−4^; KP.3 *P* = 2.44 × 10^−4^). **g**, KP.3 pseudovirus neutralization of absorbed sera from day 15 to day 45 post-KP.2 boost (ancestral *P* = 0.0045; KP.3 *P* = 0.002). Type-specific responses are shaded red. **h**, Type-specific responders subset cohort description and individual exposure histories. **i**, Pseudovirus neutralization by mock-absorbed sera from individuals with type-specific serum responses. **j**, Pseudovirus neutralization by ancestral-absorbed sera from individuals with type-specific serum responses (BA.5 *P* = 0.035; XBB.1.5 *P* = 0.035; KP.3 *P* = 0.035). **k**, Type-specific proportion of total pseudovirus neutralization titer. For the data in **a**–**g**, *n* = 14 samples. For **h**–**k**, *n* = 5 samples. Individual points represent an average of *n* = 2 technical replicates. For the data in **b**–**j**, red or black bars indicate geometric mean. For **k**, red bars indicate the median. For **b** and **c**, data were analyzed using a Wilcoxon rank-sum test. For **d**–**g** and **i**–**k**, data were analyzed using a Wilcoxon signed-rank test with a Benjamini–Hochberg correction for multiple testing. Statistical comparisons were made to the first time point (**b** and **c**), mock absorption (**d**–**g**) or ancestral strain (**i**–**k**); *P < 0.05; **P < 0.01; ***P < 0.001. All tests performed were two sided. Source data

Because repeated exposures to Omicron variants elicited new responses against mutated spike epitopes, we hypothesized that additional exposures to further mutated variants could increase the frequency of these responses. KP.2 RBD contains nine more amino acid mutations than XBB.1.5 when compared to the ancestral sequence^51^. To test how exposure to this more antigenically distant variant shaped antibody specificities, we assessed humoral responses in 14 WA1 mRNA-LNP-imprinted individuals who received the KP.2 spike-encoding mRNA-LNP booster vaccine (Fig. 5a and Extended Data Fig. 3a).

Serum binding and neutralization titers to WA1 and KP.3 variants were measured at baseline and at days 15 and 45 after boosting. Antibodies to both RBDs significantly increased over the 45 days after vaccination, suggesting robust boosting of cross-reactive immunity (Fig. 5b). We then measured neutralization titers against WA1 and KP.3 pseudoviruses, observing a significant increase in KP.3 but not WA1 titers over 45 days after boosting (Fig. 5c). It is worth noting that KP.3 neutralization titers rose from near undetectable levels to levels comparable to those elicited by the initial two-dose regimen against the ancestral strain^52^.

To determine the cross-reactivity of serum antibodies and identify variant type-specific responses after vaccination, we performed serum absorption assays on sera from days 15 to 45 after boosting. Mock (no antigen), WA1 and KP.3 spikes were coupled to magnetic beads and incubated with serum to deplete reactive antibodies. Depleted sera were then tested for binding to and neutralization of WA1 and KP.3 RBDs and pseudoviruses, respectively. WA1 spike absorption depleted almost all antibodies that bound WA1 RBD and neutralized WA1 pseudovirus, confirming the efficacy of absorption (Fig. 5d,e). KP.3 absorption only partially depleted WA1 RBD binding and nAbs, demonstrating low variant cross-reactivity (Fig. 5d,e). For KP.3 RBD binding and neutralization, WA1 spike absorption depleted almost all antibodies in 8 of 14 individuals (Fig. 5f,g), suggesting the presence of variant type-specific responses in the other 6 individuals.

As these individuals had multiple prior Omicron exposures, we sought to more fully characterize their patterns of variant reactivity (Fig. 5h). We measured the binding and neutralizing ability of serum antibodies from these individuals against prior Omicron variants. While anti-RBD binding titers were highest against the WA1 variant in these individuals, neutralization potency was greatest against the BA.5 variant (Fig. 5i and Extended Data Fig. 3b,c). The fraction of type-specific antibodies that did not bind the WA1 spike bound and neutralized BA.5 and XBB.1.5 at a similar potency as KP.3 (Fig. 5j and Extended Data Fig. 3c). Notably, the type-specific fraction of antibodies contributed the most to the total binding and neutralization titer of KP.3 compared to BA.5 and XBB.1.5; however, WA1-reactive antibodies still made up a larger fraction of the response (Fig. 5k and Extended Data Fig. 3d). Further, we observed no significant difference in KP.3 neutralization titers between individuals that did and did not have variant type-specific antibodies, suggesting that WA1-imprinted antibody responses were still driving variant neutralization across our cohort (Extended Data Fig. 3e).

These results provide evidence for strong ancestral immunological imprinting in individuals that received the KP.2 booster; however, we identified a subset of individuals where type-specific antibodies made up 25–50% of the total antibody response. Regardless of whether type-specific responses were elicited, these data highlight the importance of receiving updated booster vaccines, as few individuals had detectable immunity against currently circulating strains before vaccination.

### B cell responses after KP.2 boost

**Fig. 6:**
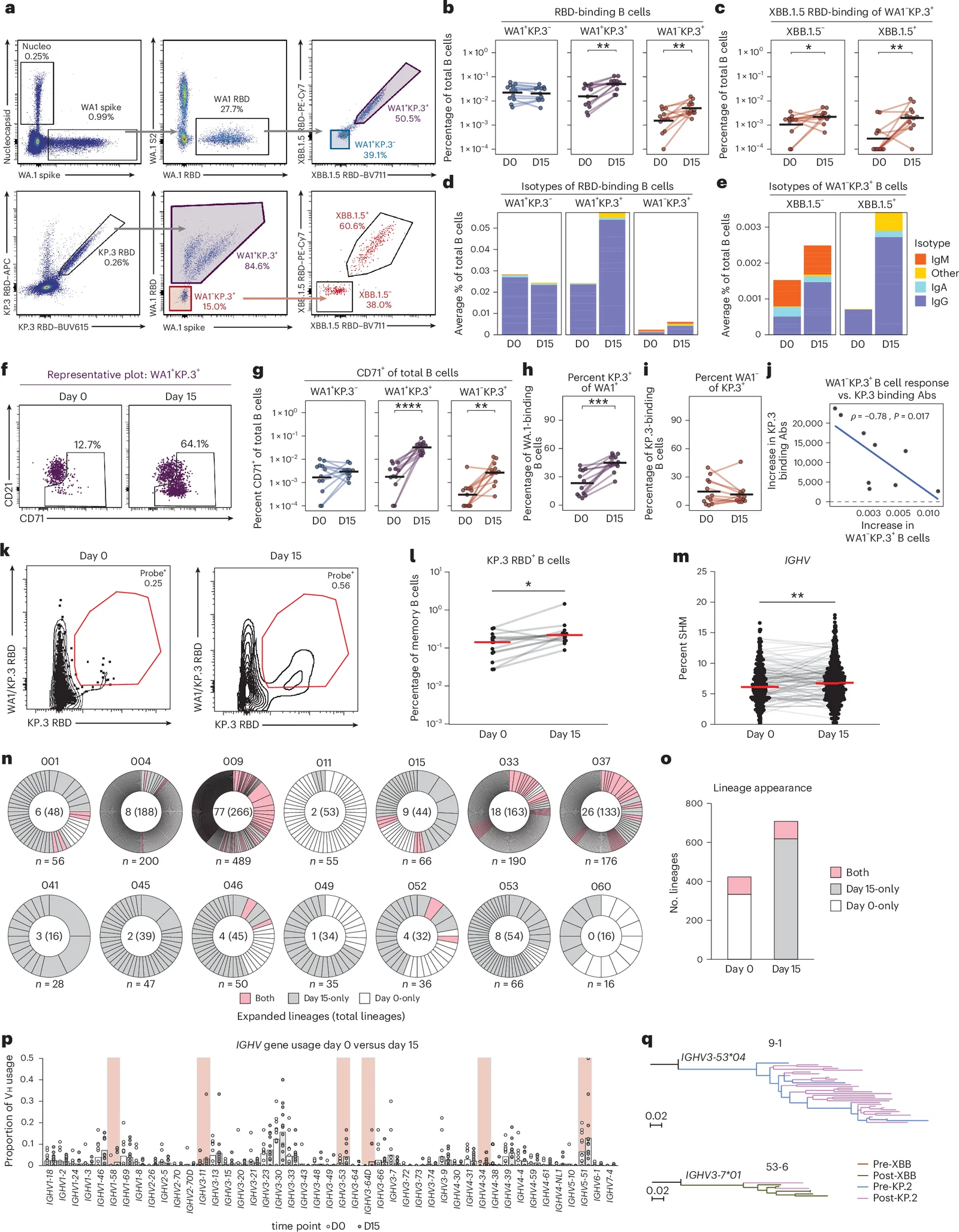
B cell response to antigenically distant KP.2 boost. **a**, Representative flow cytometry gating for antigen probe frequencies. **b**, Percentage of the total number of B cells that bind WA1 RBD only, WA1 RBD and KP.3 RBD (*P* = 0.002) or KP.3 RBD only (*P* = 0.004) before and after boost; D, day. **c**, KP.3-specific B cells that are XBB.1.5^−^ (*P* = 0.026) or XBB.1.5^+^ (*P* = 0.005) before and after boost, as a percentage of total B cells. **d**, Heavy chain isotypes of RBD probe^+^ B cells that bind WA1 RBD only, WA1 RBD and KP.3 RBD or KP.3 RBD only before and after boost. **e**, Heavy chain isotypes of KP.3^+^WA1^−^ B cells XBB.1.5^−^ or XBB.1.5^+^ before and after boost. **f**, Representative flow cytometry gating of B cell activation (CD71 staining) before and after boost. **g**, CD71^+^ B cells that bind WA1 RBD only, WA1 RBD and KP.3 RBD (*P* = 3.64 × 10^−5^) or KP.3 RBD only (*P* = 0.001) as a percentage of total B cells. **h**, Percentage of WA1 RBD-binding B cells that also bind KP.3 RBD before and after boost (*P* = 1.44 × 10^−4^). **i**, Percentage of KP.3 RBD-binding B cells that do not bind WA1 RBD before and after boost. **j**, Spearman rank correlation of the increase in frequency of KP.3 RBD-specific B cells versus the increase in antibodies that bind the KP.3 RBD as measured by ELISA. **k**, Concatenated flow cytometry plot of antigen-specific memory B cells sorted for 10x sequencing. **l**, KP.3 RBD-binding B cells as a percentage of memory B cells before and after boost (*P* = 0.01). **m**, Mean heavy chain variable percent SHM of all B cell lineages sequenced before (*n* = 423) and after (*n* = 708) boost (*P* = 0.0066). **n**, Lineage expansion and persistence in each donor. Each ring represents all B cells isolated from a donor, and each segment represents one lineage. The thickness of the segments represents the number of cells per lineage. Lineage segments are colored by time points isolated. The text inside the rings represents the number of expanded lineages (total lineages). Numbers of cells are reported below each ring. **o**, Summary of lineage appearances. **p**, Heavy chain variable gene usage frequencies before and after KP.2 boost. White and gray bars represent mean frequencies before and after boost, respectively. Dots indicate individual donor means. Red bars highlight genes with substantial mean increases after boost. **q**, Representative lineage trees. Branches are colored by time point of isolation, and branch length represents phylogenetic distance from germline. For **a**–**j**, *n* = 12 individuals. For **l** and **p**, *n* = 14 individuals. For **l** and **m**, crossbars represent the median. For the rest of the data, crossbars represent the mean. For **b**, **c**, **g**, **h** and **l**, data were analyzed by Wilcoxon signed-rank test. For **m**, data were analyzed using a Wilcoxon rank-sum test. A Benjamini–Hochberg correction was used for multiple testing; *P < 0.05; **P < 0.01; ***P < 0.001; ****P < 0.0001. All tests performed were two sided. Source data

**Extended Data Fig. 4:**
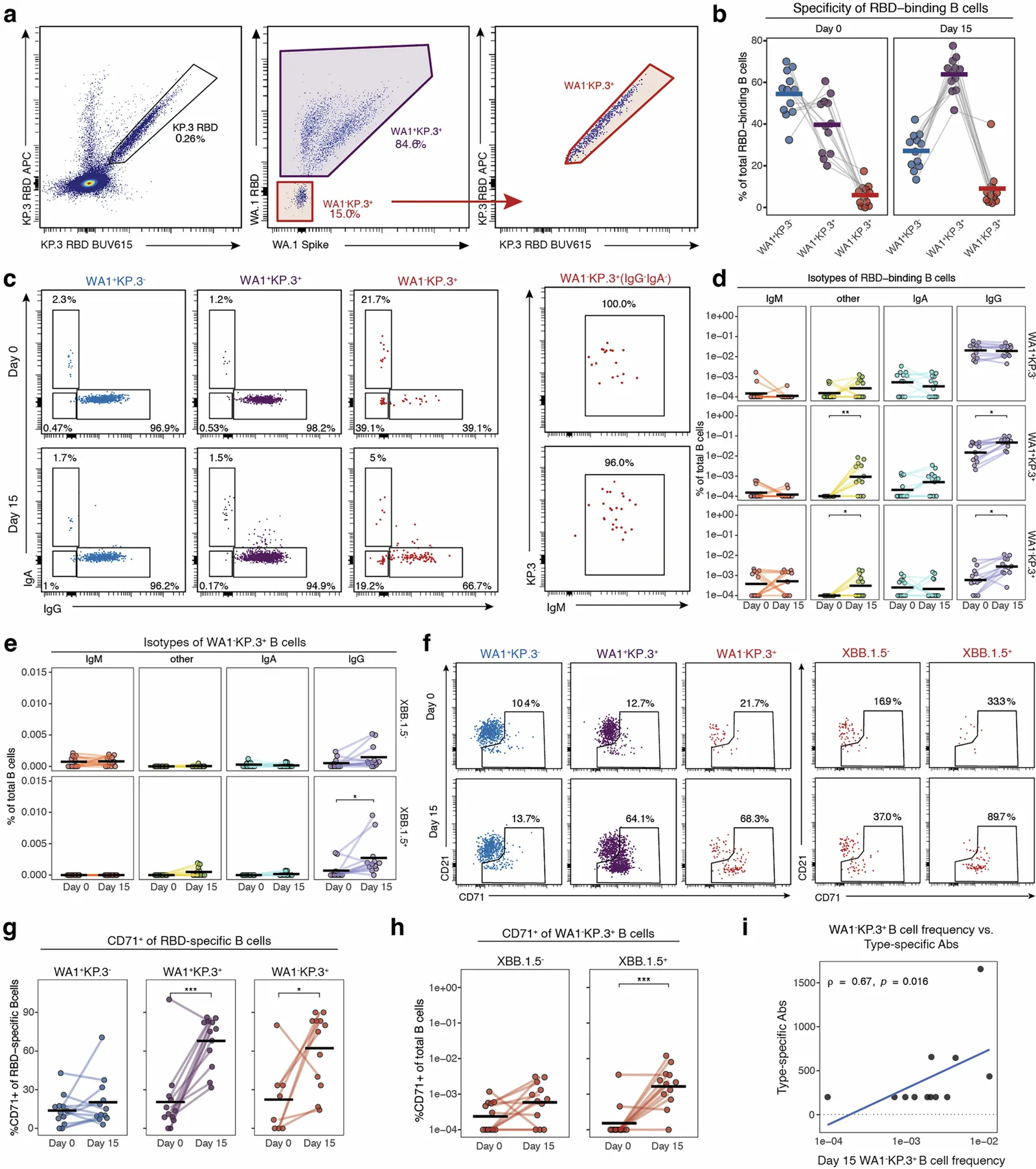
Additional B cell flow cytometry analysis. **a**) Representative flow cytometry gating for KP.3-specific RBD binding B cells. **b**) B cells that bind WA1 RBD only, WA1 RBD and KP.3 RBD, or KP.3 RBD only as a percent of total RBD binding B cells before and after boost. **c**) Representative flow cytometry gating of probe^+^ B cell heavy chain isotyping. IgA^−^IgG^−^ cells were positively gated for IgM (right). **d**) Summary of per-donor isotype frequencies in B cells that bind WA1 RBD only, WA1 RBD and KP.3 RBD, or KP.3 RBD only as a percent of total B cells before and after boost. (KP3^+^WA1^+^ other *P* = 0.0046; KP3^+^WA1^+^ IgG *P* = 0.017; KP3^+^WA1^−^ other *P* = 0.021; KP3^+^WA1^−^IgG *P* = 0.021) **e**) Summary of per-donor isotype frequencies in KP.3^+^WA1^−^ B cells that are XBB.1.5 RBD positive or negative as a percent of total B cells before and after boost (XBB.1.5^+^ other *P* = 0.049, XBB.1.5^+^ IgG *P* = 0.049). **f**) Representative flow cytometry gating of B cell activation (CD71 staining) before and after boost. **g**) Percent of RBD-specific B cells that bind WA1 RBD only, WA1 RBD and KP.3 RBD (*P* = 7.3e-4), or KP.3 RBD only (*P* = 0.011) positive for CD71 expression before and after boost. **h**) CD71^+^ KP.3 RBD specific B cells negative or positive (*P* = 7.5e-4) for XBB.1.5 RBD before and after boost as a percent of total B cells. **i**) Spearman rank correlation of day 15 KP.3-specific B cell frequencies versus KP.3 specific antibodies. For all, crossbars represent mean. For d, e, g, h, Wilcoxon signed-rank test. *p < 0.05, **p < 0.01, ***p < 0.001. All tests performed were two-sided. Source data

To assess SARS-CoV-2-specific B cell responses in the KP.2-vaccinated cohort, we performed WA1, XBB.1.5 and KP.3 RBD tetramer staining and flow cytometry of B cells before boost and 15 days after boost (Fig. 6a). We first measured frequencies of B cells reactive to WA1 only, WA1 and KP.3 or KP.3 only (Fig. 6a and Extended Data Fig. 4a). At day 15 after boost, frequencies of WA1^+^KP.3^+^ cross-reactive B cells and WA1^−^KP.3^+^ type-specific B cells significantly increased from baseline, while frequencies of WA1-only-binding B cells did not increase (Fig. 6b). As a fraction of the total RBD response within each donor, the fraction of B cells that did not bind KP.3 decreased by an average of 30%, the fraction that bound both WA1 and KP.3 increased by an average of 30%, and the fraction that bound KP.3 but not WA1 increased slightly in most donors (Extended Data Fig. 4b). Of cells that were WA1^−^KP.3^+^, frequencies of those that both did and did not bind XBB.1.5 RBD increased significantly after the boost (Fig. 6c).

We next assessed the frequencies of different heavy chain isotypes of RBD-binding B cells before and after boost (Extended Data Fig. 4c). Responses were overwhelmingly IgG, apart from a subset of WA1^−^KP.3^+^ B cells that used IgM heavy chains (Fig. 6d and Extended Data Fig. 4d). Within these WA1^−^KP.3^+^ B cells, those that did not bind XBB.1.5 RBD were 50% IgM at day 0 and 33% IgM at day 15, while those that did bind used IgG (Fig. 6e and Extended Data Fig. 4e).

We measured activation status of B cells by staining for the transferrin receptor CD71 (refs. ^53,54^; Fig. 6f and Extended Data Fig. 4f). Frequencies of cross-reactive and KP.3 type-specific B cells that were CD71^+^ significantly increased by day 15 after boost, suggesting activation in these groups, while WA1-only binders were not activated (Fig. 6g and Extended Data Fig. 4g). KP.3-only-binding B cells that did not bind XBB.1.5 had little expansion and lower frequency of CD71 expression than those that bound XBB.1.5 (Extended Data Fig. 4h). Additionally, while the fraction of WA1-binding cells that also bound KP.3 increased significantly after boost (Fig. 6h), the fraction of KP.3-binding cells that did not bind WA1 RBD did not increase (Fig. 6i).

We previously found that the individuals with greatest overall variant responses were less likely to have variant type-specific responses^40^. This observation remained true in this cohort, as we observed a negative correlation between increases in frequencies of variant type-specific B cells and total antibodies binding KP.3 RBD after boost, indicating that those with the largest overall antibody responses were less likely to have variant type-specific B cells (Fig. 6j). KP.3 type-specific antibodies were positively correlated with WA1^−^KP.3^+^ B cells (Extended Data Fig. 4i), demonstrating congruence between our two measures of variant specificity. Together, these data suggest that updated KP.2 boosting results in a significant variant-reactive response composed mainly of WA1 cross-reactive B cells. In individuals with substantial variant type-specific responses, prior Omicron exposures possibly elicited low frequencies of de novo B cells that could be subsequently recalled and expanded following KP.2 booster vaccination.

### B cell repertoire after KP.2 boost

**Extended Data Fig. 5:**
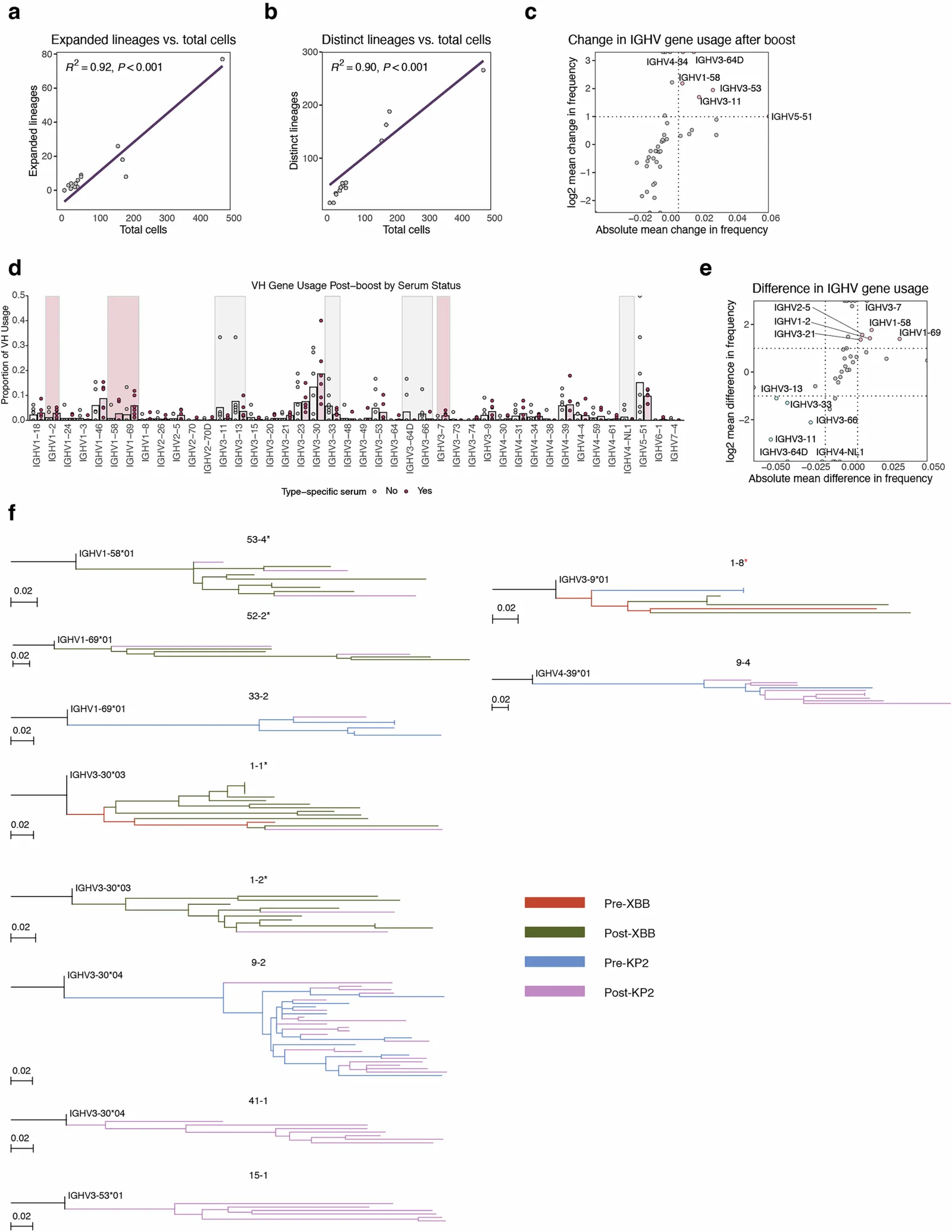
Additional B cell receptor sequence analysis. **a**) Relationship between number of expanded lineages identified versus total cells recovered per donor. Each dot represents a unique donor. **b**) Relationship between number of distinct lineages identified versus total cells recovered. Each dot represents a unique donor. Linear regression line, R^2^ value, and significance are presented. **c**) Absolute versus log2 fold-change in mean *IGHV* gene usage frequency of KP.3 RBD^+^ memory B cells after boost. **d**) Post-boost proportions of *IGHV* gene usage in unique donors by serum type-specificity as identified in Fig. 4. Each dot represents a unique donor, gray dots = fully cross-reactive serum, red dots = variant type-specific serum. Colored bars highlight genes with large mean differences between groups (red: type-specific > not type-specific, gray: not type-specific > type-specific). **e**) Absolute versus log2 fold-differences in mean *IGHV* gene usage frequency of KP.3 RBD^+^ memory B cells in donors with/donors without type-specific serum responses. **f**) Top expanded lineage trees. Lineage name consists of donor ID followed by clone ID, separated by hyphen. Branches are colored by timepoint of isolation and branch length represents phylogenetic distance from germline. Black asterisk represents validated W^+^O^+^ neutralizer, red asterisk represents validated W^−^O^+^ neutralizer. Source data

To examine changes in the variant-specific B cell repertoire following KP.2 vaccination, we sorted and sequenced 5,403 KP.3 RBD-reactive memory B cells (CD19^+^IgD^−^IgA^+^ or IgG^+^) at pre- and post-boost time points from our cohort donors (Fig. 6k). The frequency of KP.3 RBD-reactive B cells increased significantly by day 15 after boost (Fig. 6l). In total, we recovered 1,510 paired heavy and light chain sequences comprising 1,131 distinct lineages. Heavy chain variable region SHM increased significantly, providing evidence of germinal center activity (Fig. 6m). Assessing the clonal composition of variant-specific B cells before and after boost, we isolated many lineages at both time points and observed clonal expansion after boost (mean = 11.6% and range = 0–29% expanded lineages per donor; mean = 3.42 and range = 2–30 cells per expansion; Fig. 6n,o). While we identified more unique lineages after boost, this was likely a consequence of having isolated a larger absolute number of cells for this time point (Fig. 6n,o and Extended Data Fig. 5a,b).

We next measured the change in frequency of heavy chain gene usage after boost to assess preferential involvement of *IGHV* genes in the KP.2 response. We identified genes with high absolute mean and fold change in frequency after boost (>0.01 absolute change and >1 log_2_ (fold change)), including *IGHV1-58*, *IGHV3-11*, *IGHV3-53*, *IGHV3-64D*, *IGHV4-34* and *IGHV5-51* (Fig. 6p and Extended Data Fig. 5c). We also compared gene usage patterns among individuals who did and did not display variant type-specific serum responses after boost (Fig. 6a,h). We identified genes with high absolute and fold differences in frequency between groups (>0.01 absolute difference and >1 log_2_ (fold difference)), including enrichment of *IGHV1-2*, *IGHV1-58*, *IGHV1-69*, *IGHV2-5*, *IGHV3-7* and *IGHV3-21* in individuals with variant type-specific serum responses (Extended Data Fig. 5d,e). By contrast, for individuals without type-specific serum, we identified enrichment of *IGHV3-11*, *IGHV3-13*, *IGHV3-33*, *IGHV3-64D*, *IGHV3-66* and *IGHV4-NL1* (Extended Data Fig. 5d,e). While these differences were notable in both absolute and fold change measurements, none were statistically significant after adjusting for multiple comparisons.

Finally, we assessed the evolution and expansion of B cell lineages before and after vaccination. We found expansion and accumulation of SHMs in *IGHV3-53*, *IGHV3-30* and *IGHV1-69* using lineages among multiple donors (Fig. 6q and Extended Data Fig. 5f). We also observed expansion and SHM in multiple W^−^O^+^ type-specific lineages that we identified earlier after XBB exposure, suggesting possible participation of de novo lineages in responses to future variants (Fig. 6q and Extended Data Fig. 5f).

Our B cell analysis supports our serological data such that responses to the antigenically distant KP.2 spike protein were still predominantly cross-reactive to the ancestral strain. While we observed a high degree of concordance in RBD-reactive B cells across the cohort driven by memory recall, we found evidence for genetic differences in the B cell repertoire that aligned with our mAb and serological data, suggesting that additional variant-specific B cell responses were elicited after updated variant boosting.

**Fig. 7:**
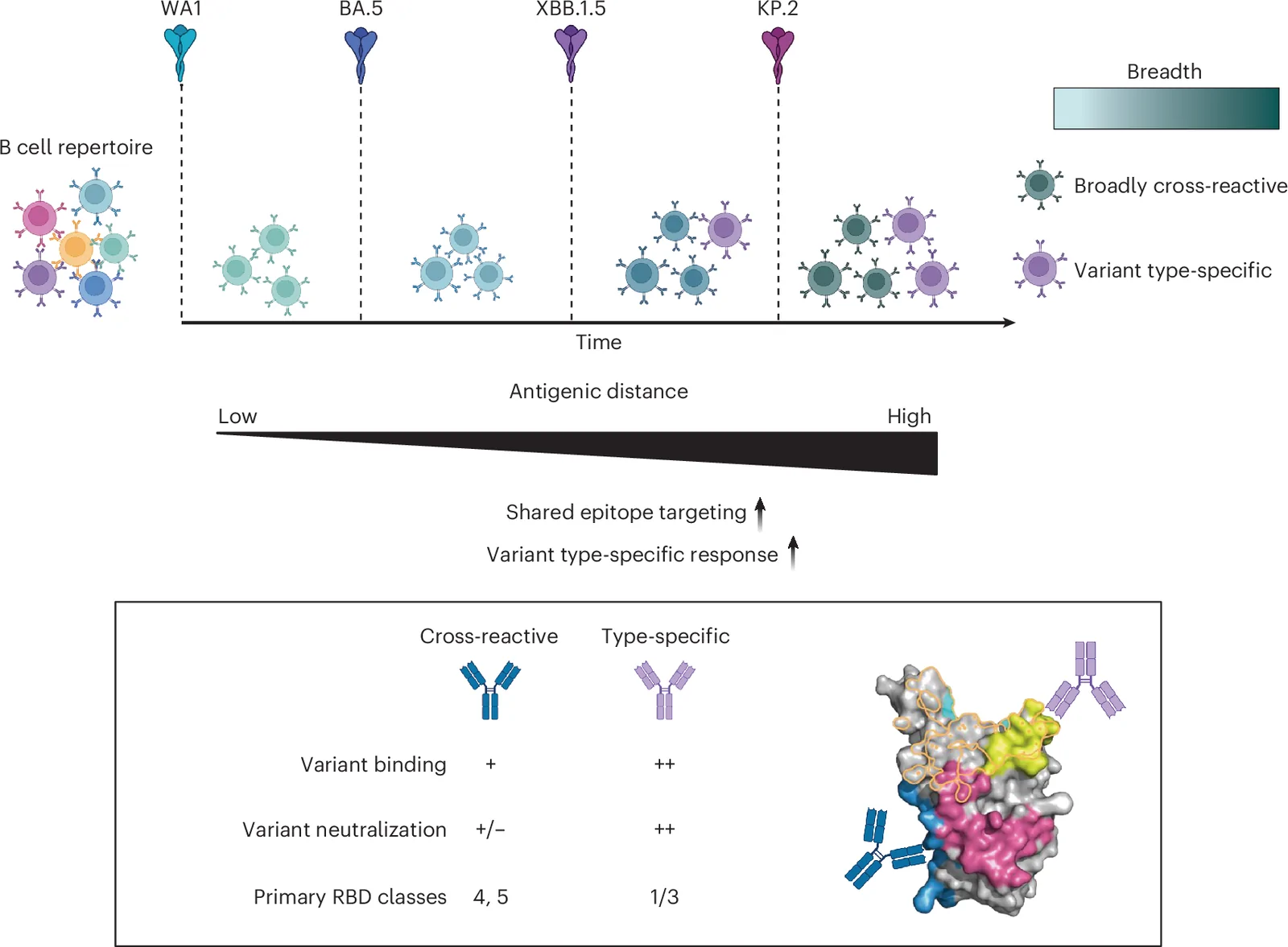
Sequential variant exposures elicit cross-reactive and type-specific antibodies. Graphical summary of findings. Figure created in BioRender; Johnston, T. https://biorender.com/nyv7tjy (2026).

In summary, our results support a model whereby successive exposures to increasingly antigenically distant variants predominantly recall ancestral cross-reactive memory B cells. The most potent antibodies from these B cells are class 1 RBD targeting, although these lose activity against future variants. Broadly, albeit less potently, neutralizing class 4 and 5 targeting antibodies comprise most of the cross-reactive response and retain function against future variants. Furthermore, as the antigenic distance of the boosting antigen from the ancestral strain increases, so does the likelihood of eliciting potent de novo variant type-specific responses against mutated epitopes on the variant RBD (Fig. 7). Together, these result in an antibody composition balancing breadth and potency, thus increasing serologic titers against both previous and future antigens.

## Discussion

In this study, we explored how pre-existing immune memory shapes the balance between recall and de novo antibody responses to sequential exposures to increasingly mutated SARS-CoV-2 variants in humans. Our principal findings were (1) potent memory recall and de novo variant type-specific responses can be elicited after Omicron exposures in ancestral-imprinted individuals; (2) variant type-specific nAbs are closer to germline, use distinct *IGHV* and *IGLV* genes, target different RBD epitopes and are generally more potent against current variants; and (3) updated KP.2 booster vaccination elicits potent nAbs and robust B cell responses. While overwhelmingly cross-reactive between ancestral and current Omicron variants, variant type-specific antibodies and B cells are elicited in some individuals.

Recent studies provide conflicting evidence regarding the necessity of matching vaccine boosts to the most recent circulating variants with the goal of eliciting de novo type-specific antibody responses. The theoretical objective would be to elicit more potent nAbs of circulating variants than recalled imprinted antibodies, thus potentially providing superior protection against infection. Although not widely observed in individuals who received mRNA vaccines, variant type-specific responses have been reported in individuals infected with Omicron variants after receiving inactivated-virus vaccines, possibly due to a combination of lower antibody titers and lower frequencies of memory B cells elicited by inactivated vaccines, as well as longer waning periods before subsequent Omicron exposures^44,55^. The Omicron-specific responses described after variant infection in inactivated vaccine-imprinted humans were composed of antibodies with notably different genetic and functional attributes from those observed in our study. We therefore speculate that primary vaccination with the same antigen but mediated by different vaccine modalities may affect the nature of the de novo antibody response at the molecular level. Despite identifying unique de novo lineages, we similarly found variant type-specific antibodies to be potent neutralizers of current and future variants. To this end, immunogen design using epitope masking techniques such as antigen resurfacing may be incorporated into boosting regimens to focus antibody responses away from poorly neutralizing epitopes and toward those that confer clinical benefit^56^.

There is also evidence for continued evolution of imprinted memory B cells over time that allows their antigen receptors to more potently neutralize current variants^39,57^. While we described a highly potent subset of ancestral cross-reactive lineages predominantly comprising *IGHV3-53* class 1 antibodies that target the RBD, we also observed populations of class 4 and 5 antibodies encoded by diverse *IGHV* genes that made up a larger proportion of the imprinted response and appear to be more resistant to mutations in current variant strains. We found that cross-reactive B cells were rapidly recalled following variant exposure and were the primary contributor to the anti-KP.2/KP.3 response regardless of whether type-specific responses were elicited, underscoring the resolute benefits of ancestral-elicited memory.

Previous work in influenza has shown that boosting with the most recent variant results in an increase in antibody titers to both past and future strains^58^. Our current work provides some mechanistic context for this observation, revealing a concurrent recall of B cells maintaining activity against both priming and boosting strains as well as the elicitation of new B cells to new epitopes on the boosting strain if sufficiently antigenically distant. Further, a portion of each group of B cells retains activity against future variants. Collectively, these results suggest that successful boosting against rapidly mutating viruses may balance antibody breadth and potency by seeking to adequately activate these B cell pools.

Our study has several limitations. We sorted cells at 15 days after exposure, and it is possible that clonal composition in the periphery could subsequently change in these individuals^37,59^. Furthermore, we specifically isolated mAbs from donors that had serological evidence for type-specific responses in our absorption assay. This does not imply that they are nonexistent in the remainder of the cohort. In our sequencing data, we found more unique lineages with more total cells recovered, assuming a representative sample size of mAb lineages for each donor, although we did not reach saturation. Finally, while we focused our study on the RBD because antibodies targeting this epitope are more frequently neutralizing^60^, full characterization of variant responses would also require studying antibodies specific to the N-terminal domain and S2.

In conclusion, the goal of updated variant boosting is to increase protective antibody titers to new virus variants to reduce severe disease in individuals and transmission at the population level. We describe a twofold benefit of boosting with progressively mutating antigens: (1) it improves virus-specific humoral immunity by engaging broad memory responses, and (2) it can elicit potent novel responses against mutated epitopes. Thus, imprinting is a fortuitous outcome of immune memory that can confer biological benefit in the context of pathogens that evolve unpredictably. It is the balance between the efficacy of the recall response and the need to elicit new variant-specific antibodies that may ultimately determine clinical outcomes.

## Methods

This research was completed in compliance with the University of Pennsylvania Institutional Review Board (IRB; 851465).

### Participant recruitment and sampling

Participants were recruited for this study under approval by the University of Pennsylvania IRB (851465) for blood sampling before and after mRNA booster vaccination or positive SARS-CoV-2 test. Written informed consent was obtained from all participants. For certain participants, baseline sampling was acquired before SARS-CoV-2 vaccination or infection, but at >1 month following their previous vaccine dose or infection. These samples are labeled as day 0 or ‘pre’ throughout the manuscript. Samples were collected on days 7, 15 and 45 based on the date of booster vaccination or the reported date of first symptom onset (IRB 851465). SARS-CoV-2 infection history was determined by self-reporting, and infecting variant was assumed by circulation proportion at the time of infection. Participants were otherwise healthy, with no self-reported history of chronic health conditions, and none were hospitalized during SARS-CoV-2 infection. Venous blood (30–100 ml) and clinical questionnaires were collected at each visit. Detailed cohort demographic information can be found in Supplementary Table 5.

### Peripheral blood sample processing

Venous blood (30–100 ml) was collected into sodium heparin tubes following standard phlebotomy procedures. Plasma was separated by centrifugation at 1,800*g* for 15 min, aliquoted and stored at −80 °C. Before binding and neutralization assays, plasma was heat inactivated at 56 °C for 30 min. After plasma removal, the remaining blood was diluted to twice the original blood volume with R1 (RPMI supplemented with 1% FBS, 2 mM L-glutamine and 100 U penicillin–streptomycin). Diluted blood was layered onto 15 ml of lymphoprep (STEMCELL Technologies) in SEPMATE tubes (STEMCELL Technologies) and centrifuged at 1,200*g* for 10 min. The peripheral blood mononuclear cell (PBMC) layer was collected, washed once with R1, pelleted and resuspended in ACK lysis buffer (Thermo Fisher). ACK lysis was performed for 5 min at room temperature and quenched with R1. PBMCs were filtered through a 70-µm strainer, counted using a Countess automated counter (Thermo Fisher) and cryopreserved in aliquots of 1 × 10^7^ cells in 90% FBS/10% DMSO at −80 °C.

### Flow cytometry-assisted sorting of single B cells

Antigen-specific B cells were sorted with biotinylated RBD streptavidin (SA)–fluorophore conjugate probes. All reagents can be found in Supplementary Table 2. Probes were preprepared by incubating biotinylated RBDs with fluorescently labeled SA overnight. Each probe included a 5 μg:2 μg ratio of biotinylated RBD:fluorescently labeled SA. WA1 RBD was conjugated to APC-SA and BV785-SA, and XBB.1.5 RBD was conjugated to BV605-SA and PECy7-SA (Biolegend). On the day of sorting, 10 × 10^6^ PBMCs were thawed from cryopreservation and washed with R10 medium (RPMI + 10% FBS + 5% penicillin–streptomycin) + 0.05% benzonase (Millipore). Cells were stained with Ghost Violet 510 Viability Dye (Tonbo; 1:500) at room temperature for 10 min and washed in BSA buffer (BD Biosciences) before surface and probe master mix was added. Each reaction contained a 50-μl surface master mix of anti-CD3–FITC, anti-CD14–FITC, anti-CD16–FITC, anti-IgD–BB700, anti-CD19–BV421, anti-CD38–BUV661, anti-IgG–Ax700 and anti-IgA–PE (1:200 final dilution for all antibodies). A 50-μl probe mix of WA1 RBD–APC, WA1 RBD–BV785, XBB.1.5 RBD–BV605, XBB.1.5 RBD–PECy7 and 5 μM free biotin (Avidity) was added and mixed thoroughly. This reaction was incubated at 4 °C for 30 min. Cells were then washed, resuspended in 100 μl of R10 and immediately sorted. All washes were performed by spinning at 500*g* for 5 min. Single cells were sorted using a BD FACSymphony A5 sorter (BD Biosciences) into 96-well plates with wells containing 5 μl of Qiagen TCL Buffer (Qiagen) + 1% β-mercaptoethanol (Sigma-Aldrich) and immediately stored at –80 °C. For 10x sorts, WA1 RBD and KP.3 RBD were both conjugated to TotalSeqC SA-APC–oligonucleotide (Biolegend), and KP.3 RBD was conjugated to SA-PECy7 (Biolegend). One microliter of TotalSeqC anti-human hashtag (Biolegend) was added to each sample during surface staining. Data were analyzed using FlowJo v10.

### mAb isolation and expression

To isolate heavy and light chain variable regions from single-sorted B cells and express their receptors as mAbs, we performed RATP-Ig as previously described^45^. Briefly, RNA was extracted from single B cells, and 5′ rapid amplification of cDNA ends was performed to synthesize and amplify cDNA. A second round of PCR was performed to amplify DNA for IgG, IgA, IgK and IgL sequences. Immunoglobulin enrichment products were split for library preparation and assembly into antibody expression cassettes for heavy and light chain antibody fragments. Expression cassettes were co-transfected into Expi-293 cells (Thermo Fisher, A14527) and shaken using a 19-mm orbital shaker at 1,000 rpm (21*g*) and 37 °C with 8% CO_2_ for 5 days before clarification by centrifugation at 3,000*g* for 30 min.

### Single-cell V(D)J sequencing

All sequences can be found in Supplementary Table 1.

### RATP-Ig

Immunoglobulin enrichment products were prepared into DNA libraries using the Illumina Nextera XT library preparation kit (Illumina). Illumina-ready libraries were sequenced on a NextSeq 2000 (Illumina) using a P1 300 cycle kit (Illumina) for paired-end 2 × 150 base pair reads to obtain V(D)J sequences.

### 10x

To analyze the V(D)J repertoires in sorted single B cells, a cell suspension was loaded on the 10x Genomics Chromium Instrument with reagents from the Next GEM Single Cell 5′ Kit v2 (10x Genomics, 1000263) to generate gel bead-in-emulsions. Following the manufacturer’s protocol, gel bead-in-emulsion reverse transcription was performed, followed by purification and amplification of the total cDNA. Heavy and light chains were amplified using immunoglobulin-specific 3′ primers with the addition of Illumina sequences. The Illumina-ready libraries were sequenced on a NextSeq 2000 (Illumina) using a P1 600 cycle kit for paired-end 2 × 300 base pair reads. For hashing data, the 10x Genomics protocol for sequencing of surface DNA was followed.

### V(D)J analysis

Analysis was performed as previously reported^45^. Briefly, sequencing reads were demultiplexed, and V(D)J sequences were assembled using BALDR^62^. Sequences were then filtered using a custom script (https://github.com/scharch/filterBALDR) to remove incomplete sequences. Filtered sequences were annotated with SONAR v4.2 (ref. ^63^). The OGRDB database was used to identify and annotate V, D and J genes using IMGT nomenclature^64,65^.

For sequences generated from 10x Chromium, reads from the hashing libraries were analyzed with Cell Ranger (10x Genomics). The resulting count matrix was loaded into Seurat for downstream analysis, and sample identities were assigned using the HTODemux function^66^. For the V(D)J libraries, paired-end reads were subjected to preprocessing, merging and annotation in SONAR under single-cell mode with unique molecular identifier detection enabled, using default settings. For all analyses, we discarded any nonproductive rearrangements and cells with multiple heavy or light chains. To assemble lineage trees, sequences were analyzed using SONAR v4 in single-cell mode^63^. Trees were made from concatenated heavy and light chain sequencing data using IgPhyML^67^ in SONAR.

### Total IgG quantitation

Total IgG was quantified using a Valita Titer Assay (2.5–100 mg l^−1^) and 384-well plates (ValitaCell, VAL013), according to the manufacturer’s instructions, on an integrated automation platform consisting of a Biomek liquid handler from Beckman Coulter and a Molecular Devices Paradigmor i3x Multimode reader. Undiluted RATP-Ig supernatants and a serially diluted purified IgG reference standard (1–150 µg ml^−1^) were transferred at 40 µl per well to Valita Titer plates (prewetted with PBS at 40 µl per well), incubated for 15 min at room temperature and read using fluorescence polarization (FP-FLUO detection cartridge, Molecular Devices). Concentrations were interpolated relative to the reference standard. Supernatants below the lower limit of quantification were considered 0.5 µg ml^−1^ for calculations. Concentrations can be found in Supplementary Table 1.

### Supernatant binding

Supernatants were screened for binding to the SARS-CoV-2 RBDs of WA1, BA.5, XBB.1.5 and KP.3 using an electrochemiluminescence immunoassay and Meso Scale Diagnostic (MSD) U-PLEX 96-well plates (K15235N). Biotinylated RBDs (200 µl at 10 µg ml^−1^) were individually incubated with MSD U-PLEX linkers (linkers 1–4 at 300 µl) for 30 min at room temperature. The reaction was quenched for 30 min with MSD Stop Solution and brought to the manufacturer’s recommended volume for coating. The quenched RBD/U-PLEX linker coating solution was added to plates (50 µl per well), with shaking at 900 rpm (1*g*) for 1 h. Plates were washed, and supernatants were diluted 1:100 in MSD Diluent 100 (25 µl per well), with shaking at 900 rpm (1*g*) for 1 h. Plates were washed, and MSD Sulfo-Tag anti-human IgG detection antibody at 1× (D21ADF; 25 µl per well) was added, with shaking at 900 rpm (1*g*) for 1 h. Plates were again washed, and MSD Read Buffer B (R60AM; 150 µl per well) was added to each well. Plates were read on an MSD S600 Reader. Electrochemiluminescence signals to each respective RBD-coated spot in a well was used as a measure of binding. The ECL cutoff for antigen binding was >10,000. MSD values for mAbs can be found in Supplementary Table 1.

### mAb pseudovirus neutralization

mAb supernatant neutralization activity was measured as previously described^68^ using pseudoviruses expressing SARS-CoV-2 D614G, BA.5, XBB.1.5 and KP.3 spikes. Briefly, SARS-CoV-2 lentiviral pseudotyped neutralization assays were performed on an integrated automation platform consisting of a Biomek liquid handler from Beckman Coulter, an ambient temperature labware hotel (Thermo Scientific), a 37 °C incubator (Thermo Scientific) and a Molecular Devices i3x Multimode reader. The automated assay methods were operated using Beckman Coulter SAMI EX software (version 5.0). To screen, supernatants were diluted to 1:20 in D10 culture medium (10% FBS, DMEM and 0.3 µl ml^−1^ puromycin). To generate IC_50_ curves, supernatants were diluted starting at 1:20 and then serially diluted fourfold (seven times) in D10 culture medium. SARS-CoV-2 S-pseudotyped viruses were diluted in D10 and added (30 µl per well) into tissue culture plates containing the diluted samples (30 µl per well), followed by a 45-min incubation at 37 °C with 5% CO_2_. Human 293T ACE2 reporter cells were added at a concentration of 10,000 cells per well in 20 µl into virus/sample tissue culture plates, followed by a 72-h incubation at 37 °C with 5% CO_2_. Plates were developed by removing 50 µl of culture medium from the plates and adding 30 µl of luciferase substrate (Revvity Britelite Plus, 6066769). The luminescence signal (relative luminescence units (RLU)) was measured using a i3x Multimode reader. Neutralization percentage of the sample was determined by normalization of the test sample RLU to the RLU of virus and cell control wells with the following calculation: percentage = [(test wells – average of cell control wells) – (average of virus control wells – average cell control wells)] ÷ (average virus control wells – average cell control wells) × 100. For serially diluted samples, the neutralization curve fit was generated on a NAB analysis module on the Labkey web-based server with five-parameter nonlinear regression. IC_50_ measurements were performed in duplicate. nAb titers are expressed as the reciprocal of the serum dilution required to reduce RLU by 50% and are reported as inhibition concentration. The cutoff for mAb neutralization was >50%. Antibodies were classified as W^+^O^−^ if WA1 was >50% and all Omicron variants were <50%. Antibodies were classified as W^+^O^+^ if WA1 and any Omicron variant was >50%. Antibodies were classified as W^−^O^+^ if WA1 was <50% and any Omicron variant was >50%. Neutralization values for mAbs can be found in Supplementary Table 1.

### High-throughput surface plasmon resonance

Classical epitope binning experiments were performed on the Carterra LSA XT. Five fourfold dilutions (10,000 ng ml^−1^ to 39.5 ng ml^−1^) of reference mAbs were amine coupled to an HC30M chip (Carterra). mAb coupling performance was assessed with regeneration scouting using 50 nM XBB.1.5 RBD. For binning, cycles included 1-min baseline, 50 nM XBB.1.5 RBD injection with 5 min of association time and 10 μg ml^−1^ competitor mAb injection with 5 min of association time, followed by a 1-min dissociation time and two 30-s regenerations with 10 mM glycine pH 2.0 (Carterra). Response units measurements were acquired at 25 °C. Analysis was performed using Carterra Epitope Software v.1.9.2.4505 (Carterra). Consistency across mAb dilutions was determined, and consistent chip regions of interest were used across experiments. Our class 2 reference mAb did not display consistency across regions of interest and experiments, so data were excluded from binning. Epitope determinations can be found in Supplementary Table 1.

### Deep mutational scanning

Deep mutational scanning was performed as described previously^49^. In total, 1.5 million transcription units of XBB.1.5 full-spike deep mutational scanning libraries were mixed with non-neutralizable standard and incubated with increasing antibody concentrations for 45 min at 37 °C. The following concentrations were used: 0.2, 0.5 and 1.5 µg ml^−1^ for 018-1; 0.4, 1.3 and 3.9 µg ml^−1^ for 053-6; 0.8, 2.5 and 7.6 µg ml^−1^ for 053-3; 0.5, 1.5 and 4.45 µg ml^−1^ for 043-1 and 0.7, 2 and 6.1 µg ml^−1^ for 052-3. These concentrations reached greater than 70% of the virus library neutralization as measured by deep mutational scanning. After incubation, medium-ACE2 cells^69^ were infected with virus–antibody mixtures, and 15 h after infection, non-integrated viral genomes were recovered and prepared for Illumina sequencing as described previously^49^. Non-neutralizable standard was used to calculate escape caused by each mutation in the deep mutational scanning library. This analysis used a biophysical model implemented in polyclonal (v6.16)^70^. Experiments were performed using two biological library replicates. The full computational analysis pipeline for deep mutational scanning data is available at https://github.com/dms-vep/SARS-CoV-2_XBB.1.5_spike_DMS_NIH_Penn_mAbs.

### Negative-stain electron microscopy analysis

Fabs were produced and purified using a Pierce Fab Preparation Kit (Thermo Fisher Scientific). Each Fab was mixed with SARS-CoV-2 variant XBB.1.5 spike at a 3:1 molar ratio, and the mixture was diluted in a negative-stain buffer (10 mM HEPES (pH 7.0) and 150 mM NaCl) to achieve a spike trimer concentration of approximately 0.02 mg ml^−1^. In total, 4.8 ml of the diluted Fab–spike mixture was pipetted onto freshly glow-discharged carbon-coated grids, and the liquid was wicked away with a piece of filter paper. The grid was rinsed three times with 4.8 ml of the negative-stain buffer and stained with 0.75% uranyl formate for 30 s. The excess staining solution was wicked away, and the grid was air dried. Data were collected using a Talos F200C transmission electron microscope (Thermo Fisher Scientific) operated at 200 kV and equipped with a Ceta camera at a nominal magnification of ×57,000, corresponding to a pixel size of 2.53 Å. Particle picking, two-dimensional classification and three-dimensional reconstruction were performed using CryoSparc 4.6 (ref. ^71^). Visualization, docking, molecular analysis and figure preparation were performed using UCSF ChimeraX^72^. AlphaFold 3 (ref. ^73^) was used to predict the structural models of the Fabs. Structural models of spike (PDB: 8A94)^61^ and Fab were docked into negative-stain three-dimensional maps with rigid-body fitting of individual domains. The interface between the RBD and Fab was examined to estimate the epitope of each mAb.

### Serum antibody binding

Antigen-specific ELISAs were performed as previously described^40^. High-binding plates (Thermo Fisher) were coated with antigen diluted to 2 μg ml^−1^ in DPBS overnight at 4 °C. The following day, plates were washed three times with PBS + 0.1% Tween-20 (PBS-T) and blocked for 1 h at room temperature with 200 μl of blocking buffer (DPBS + 3% milk powder + 0.1% Tween-20). After blocking, plates were washed three times with PBS-T, and 50 μl of serum that was serially diluted in dilution buffer (DPBS supplemented with 1% milk and 0.1% Tween-20) was added to each well. Plates were incubated for 2 h at room temperature and then washed three times with PBS-T. In total, 50 μl of goat anti-human IgG horseradish peroxidase conjugate (Jackson) was added to each well and allowed to incubate at room temperature for 1 h. After a final set of three PBS-T washes, 50 μl of SureBlue 3,3′,5,5′-tetramethylbenzidine substrate (SeraCare) was added to each well. Plates were incubated in the dark for 5 min before stopping with 25 μl of 250 mM HCl. Plates were read at an optical density at 450 nm using a SpectraMax plate reader (Molecular Devices). Serum antibody titers were calculated from a standard curve of serially diluted pooled serum (starting dilution of 1:100). Reciprocal end point titers were calculated using GraphPad Prism (v.10.1.1) four-parameter logistic regression. PBS-coated plates were included for each sample dilution plate to account for nonspecific binding. Antibody titers for each sample were measured in two technical replicates. ELISA data are available in Supplementary Table 3.

### Serum pseudovirus neutralization

Focus reduction neutralization titers were determined as previously described^40^. Briefly, 96-well collagen-coated plates were seeded with 2.5 × 10^4^ TMPRSS2-expressing VeroE6 cells per well the night prior. The next day, heat-inactivated sera or postabsorption sera were serially diluted twofold and mixed with 200–300 focus-forming units per well of VSVΔG-RFP SARS-CoV-2 pseudotype viruses. Medium used for serum and virus dilution contained 600 ng ml^−1^ anti-VSV-G 1E9F9 to ensure neutralization of any VSV-G carryover virus from pseudotype production. The serum–virus mixture was incubated for 1 h at 37 °C before being plated on VeroE6 TMPRSS2 cells. After 22 h of incubation at 37 °C, plates were washed with DPBS and fixed with 4% paraformaldehyde. The spots were visualized and counted on an S6 FluoroSpot Analyzer (CTL). The half-maximum focus reduction neutralization titer was determined as the greatest serum dilution at which focus count was reduced by at least 50% relative to control wells infected with only pseudotype viruses in the absence of human serum. Half-maximum focus reduction neutralization titers for each sample were measured in two technical replicates. Neutralization data can be found in Supplementary Table 4.

### Serum absorption

Sera were absorbed following a previously reported protocol^40^. Briefly, carboxyl magnetic beads (Ray Biotech) were incubated with the antigen of choice for 2 h at 4 °C with shaking. Antigen-conjugated beads were washed with 50 mM Tris (pH 7.4) and incubated for 15 min. Beads were then washed with PBS supplemented with 0.1% BSA and 0.05% Tween-20 a total of four times. Beads were resuspended in PBS supplemented with 0.1% BSA and 0.05% Tween-20. For absorption, antigen-coupled beads were mixed with diluted serum for a final serum dilution of 1:50. The serum–bead mixture was shaken for 1 h at 1,000 rpm (2*g*). The mixture was then placed on a magnet, and beads were allowed to separate before transferring depleted serum to a clean tube.

### Antigen-specific B cell phenotyping

Antigen-specific B cells were detected using biotinylated proteins in combination with different SA–fluorophore conjugates as previously described^52^. All reagents are listed in Supplementary Table 2. For each sample, biotinylated proteins were multimerized with fluorescently labeled SA for 1.5 h at 4 °C at the following ratios, all of which are ∼4:1 molar ratios calculated relative to the SA-only component irrespective of fluorophore: 200 ng of full-length WA1 spike protein was mixed with 20 ng of SA–BV421, 25 ng of WA1 RBD was mixed with 12.5 ng of SA–PE, 25 ng of XBB.1.5 RBD was mixed with 12.5 ng of SA–BV711, 25 ng of XBB.1.5 RBD was mixed with 12.5 ng of SA–PE-Cy7, 25 ng of KP.3 RBD was mixed with 12.5 ng of SA–BUV615, 25 ng of KP.3 was mixed with 12.5 ng of SA–APC, and 50 ng of nucleocapsid protein was mixed with 14 ng of SA–BV605. In total, 12.5 ng of SA–PE-Cy5 was used as a decoy probe without biotinylated protein to gate out cells that nonspecifically bound SA. All experimental steps were performed in a 50:50 mixture of PBS + 2% FBS and Brilliant buffer (BD Biosciences). Antigen probes were prepared individually and combined after multimerization with 5 μM free D-biotin (Avidity) to minimize potential cross-reactivity between probes. For staining, 2 × 10^6^–10 × 10^6^ PBMCs per sample were thawed from cryopreservation and prepared in a 96-well round-bottom plate. Cells were first stained with Fc block (BioLegend; 1:200) and Ghost Violet 570 Viability Dye (Biolegend; 1:500) for 15 min at 4 °C. Cells were then washed and stained for 1 h at 4 °C with 50 μl of antigen probe master mix containing the above probes combined immediately before staining. Following this incubation period, cells were washed again and stained with anti-CD27–BUV395 (1:200), anti-CD3–BUV563 (1:200), anti-CD38–BUV661 (1:1,000), anti-IgD–BV480 (1:50), anti-CD19–BV750 (1:100), anti-IgA–FITC (1:400), anti-CD21–PE-CF594 (1:500), anti-IgG–AF700 and anti-CD71–APC-H7 (1:50) for 30 min at 4 °C. After surface staining, cells were washed and fixed in 1× Stabilizing Fixative (BD Biosciences) overnight at 4 °C. Samples were run on a Cytek Aurora (Cytek), and data were analyzed using OMIQ.

### Statistical analysis

All statistical analyses were performed in R (v.4.3.3) unless specifically indicated otherwise in the Methods. Shapiro–Wilk testing was performed to test normality of data distributions. Wilcoxon rank-sum, Wilcoxon signed-rank, Spearman rank correlation and linear regression usage is indicated in the figure legends. All tests performed were two sided. Crossbars represent median, mean or geometric mean, as indicated in the figure legends. Multiple testing analyses performed are described in the figure legends.

### Reporting summary

Further information on research design is available in the Nature Portfolio Reporting Summary linked to this article.

## Online content

Any methods, additional references, Nature Portfolio reporting summaries, source data, extended data, supplementary information, acknowledgements, peer review information; details of author contributions and competing interests; and statements of data and code availability are available at 10.1038/s41590-026-02613-4.

## Supplementary information

Supplementary Information Supplementary Tables 2 and 5.

Reporting Summary

Supplementary Tables 1, 3 and 4 mAb sequence and functional data, serum ELISA data and serum neutralization data.

## Source data

Source Data Fig. 1 Statistical source data.

Source Data Fig. 2 Statistical source data.

Source Data Fig. 3 Statistical source data.

Source Data Fig. 5 Statistical source data.

Source Data Fig. 6 Statistical source data.

Source Data Extended Data Fig. 1 Statistical source data.

Source Data Extended Data Fig. 2 Statistical source data.

Source Data Extended Data Fig. 3 Statistical source data.

Source Data Extended Data Fig. 4 Statistical source data.

Source Data Extended Data Fig. 5 Statistical source data.

## Data Availability

Supplementary Information is available for this paper. Sequencing data were deposited to the Gene Expression Omnibus under accession code GSE325987. Source data are provided with this paper.

## Code availability

No custom code was created for this manuscript. The full computational analysis pipeline for deep mutational scanning data is available at https://github.com/dms-vep/SARS-CoV-2_XBB.1.5_spike_DMS_NIH_Penn_mAbs. R scripts used to create the figures are available upon author request.
