# Supplementary material for: Potent type-specific de novo antibodies complement broadly reactive imprinted antibodies in immune responses to SARS-CoV-2 variants
Source: Nat Immunol. 2026 Aug 14;27(9):1874–86. doi: 10.1038/s41590-026-02613-4 (PMC13506328; doi:10.1038/s41590-026-02613-4)
Supplement: Supplementary file 1 [file 41590_2026_2613_MOESM1_ESM.pdf]

nature immunology

Supplementary information https://doi.org/10.1038/s41590-026-02613-4

Potent type-specific de novo antibodies complement broadly reactive imprinted antibodies in immune responses to SARS-CoV-2 variants

In the format provided by the authors and unedited

Table S2. Key Resources Table

Reagent	Source	Catalog Number
**Antibodies**		
BUV563 anti-CD3	BD Biosciences	Cat#748569
BV750 anti-CD19	Biolegend	Cat#302262
BV421 anti-CD19	Biolegend	Cat#302233
BUV395 anti-CD27	BD Biosciences	Cat#563815
BUV661 anti-CD38	BD Biosciences	Cat#612969
APC-H7 anti-CD71	BD Biosciences	Cat#563671
AF700 anti-IgG	Biolegend	Cat#337220
AF700 anti-IgG	BD	Cat#561296
FITC anti-IgA	Miltenyi	Cat#130-113-475
FITC anti-CD3	Biolegend	Cat#300406
FITC anti-CD14	Biolegend	Cat#325604
FITC anti-CD16	Biolegend	Cat#360716
BB700 anti-IgD	BD Biosciences	Cat#566538
BV480 anti-IgD	BD Biosciences	Cat#566138
PE/Dazzle 594 anti-CD21	Biolegend	Cat#354922
BV510 anti-IgM	BD Biosciences	Cat#563113
RB545 anti-CD20	BD Biosciences	Cat#569757
PE anti-IgA	Miltenyi	Cat#130-116-878
TotalSeq™-C0251 anti-human Hashtag 1 Antibody	Biolegend	Cat#394661
TotalSeq™-C0252 anti-human Hashtag 2 Antibody	Biolegend	Cat#394663
TotalSeq™-C0253 anti-human Hashtag 3 Antibody	Biolegend	Cat#394665
TotalSeq™-C0254 anti-human Hashtag 4 Antibody	Biolegend	Cat#394667
TotalSeq™-C0255 anti-human Hashtag 5 Antibody	Biolegend	Cat#394669
TotalSeq™-C0256 anti-human Hashtag 6 Antibody	Biolegend	Cat#394671
TotalSeq™-C0257 anti-human Hashtag 7 Antibody	Biolegend	Cat#394673
TotalSeq™-C0258 anti-human Hashtag 8 Antibody	Biolegend	Cat#394675
TotalSeq™-C0259 anti-human Hashtag 9 Antibody	Biolegend	Cat#394677
TotalSeq™-C0260 anti-human Hashtag 10 Antibody	Biolegend	Cat#394679
TotalSeq™-C0296 anti-human Hashtag 11 Antibody	Biolegend	Cat#328941
TotalSeq™-C0262 anti-human Hashtag 12 Antibody	Biolegend	Cat#394683
TotalSeq™-C0263 anti-human Hashtag 13 Antibody	Biolegend	Cat#394685
TotalSeq™-C0264 anti-human Hashtag 14 Antibody	Biolegend	Cat#394687
goat anti-human IgG HRP conjugate	Jackson Immunoresearch	Cat#109-035-006
anti-VSV-G antibody 1E9F9		
SULFO-TAG Anti-Human IgG Antibody	MSD	Cat#D21ADF-3
**Chemicals, peptides and recombinant proteins**		
SARS-CoV-2 Biotyinlated Full Length Spike	R&D Systems	Cat#AVI10549-050
SARS-CoV-2 Biotyinlated RBD (WA.1)	Acro Biosystems	Cat#SPD-C82E9-25ug
SARS-CoV-2 Biotyinlated RBD (XBB.1.5)	Acro Biosystems	Cat#SPD-C82Q3-25ug
SARS-CoV-2 Biotyinlated RBD (KP.3)	In-house	
SARS-CoV-2 Biotyinlated N-Terminal Domain	Sino Biological	Cat#40591-V49H-B
SARS-CoV-2 Biotyinlated S2	Acro Biosystems	Cat#S2N-C52E8-25ug
SARS-CoV-2 Biotyinlated Nucleocapsid	R&D Systems	Cat#BT10474-050

BV421 Streptavidin	Biolegend	Cat#405226
BV605 Streptavidin	Biolegend	Cat#405229
BV711 Streptavidin	BD Biosciences	Cat#563262
BV786 Streptavidin	BD Biosciences	Cat#563858
BUV615 Streptavidin	BD Biosciences	Cat#613013
BUV737 Streptavidin	BD Biosciences	Cat#612775
BB515 Streptavidin	BD Biosciences	Cat#564453
PE Streptavidin	Biolegend	Cat#405203
PE-Cy7 Streptavidin	Biolegend	Cat#405206
APC Streptavidin	Biolegend	Cat#405207
TotalSeq™-C0958 APC Streptavidin	Biolegend	Cat#405293
TotalSeq™-C0959 APC Streptavidin	Biolegend	Cat#405159
Zombie Yellow Viability Dye	Biolegend	Cat# 423104
Human TruStain FcX (Fc Receptor Blocking Solution)	Biolegend	Cat#422302
Ghost Dye 510 Viability Dye	Tonbo	Cat#13-0870-T100

Table S5. Cohort Demographics

	Type-specific Responders	KP.2 Vaccination
**N**	5	14
**Age**		
age min	25	25
age max	31	71
age mean	27.4	39.9
**Sex**		
M	2	6
F	3	8
**Race**		
White	4	11
Black	1	1
Asian	0	2
**Ethnicity**		
NHL	0	1
HL	0	0
