# Supplementary material for: Potent type-specific de novo antibodies complement broadly reactive imprinted antibodies in immune responses to SARS-CoV-2 variants
Source: Nat Immunol. 2026 Aug 14;27(9):1874–86. doi: 10.1038/s41590-026-02613-4 (PMC13506328; doi:10.1038/s41590-026-02613-4)
Supplement: Supplementary file 2 [file 41590_2026_2613_MOESM2_ESM.pdf]

nature portfolio

Corresponding author(s): Daniel C. Douek

Last updated by author(s): Jun 25, 2026

Reporting Summary

Nature Portfolio wishes to improve the reproducibility of the work that we publish. This form provides structure for consistency and transparency in reporting. For further information on Nature Portfolio policies, see our Editorial Policies and the Editorial Policy Checklist.

Statistics

For all statistical analyses, confirm that the following items are present in the figure legend, table legend, main text, or Methods section.

n/a	Confirmed	
☐	☒	The exact sample size (*n*) for each experimental group/condition, given as a discrete number and unit of measurement
☐	☒	A statement on whether measurements were taken from distinct samples or whether the same sample was measured repeatedly
☐	☒	The statistical test(s) used AND whether they are one- or two-sided<br>*Only common tests should be described solely by name; describe more complex techniques in the Methods section.*
☒	☐	A description of all covariates tested
☐	☒	A description of any assumptions or corrections, such as tests of normality and adjustment for multiple comparisons
☐	☒	A full description of the statistical parameters including central tendency (e.g. means) or other basic estimates (e.g. regression coefficient) AND variation (e.g. standard deviation) or associated estimates of uncertainty (e.g. confidence intervals)
☐	☒	For null hypothesis testing, the test statistic (e.g. *F*, *t*, *r*) with confidence intervals, effect sizes, degrees of freedom and *P* value noted<br>*Give P values as exact values whenever suitable.*
☒	☐	For Bayesian analysis, information on the choice of priors and Markov chain Monte Carlo settings
☒	☐	For hierarchical and complex designs, identification of the appropriate level for tests and full reporting of outcomes
☐	☒	Estimates of effect sizes (e.g. Cohen's *d*, Pearson's *r*), indicating how they were calculated

Our web collection on statistics for biologists contains articles on many of the points above.

Software and code

Policy information about availability of computer code

Data collection

Flow cytometry data were collected using FACSDiva v9.5.1 (BD) or SpectroFlo (Cytek). The automated assay methods were operated using Beckman Coulter SAMI EX software (version 5.0). Epitope binning data was collected using LSA navigator v1.7.4. Illumina sequencing was collected using Nextseq software v1.7.1.46395. 10x Genomics sequencing was collected using chromium controller control board FW v4.00, b01 and display board FW v4.00, b01. Serum ELISA data was collected using SoftMax Pro 7.1.2 and FRNT data was collected using ImmunoSpot 7.0 Software.

Data analysis

No new algorithms were developed for this study.

Sequencing:
sequencing reads were demultiplexed and V(D)J sequences were assembled using BALDR. Sequences were then filtered using a custom script (https://github.com/scharch/filterBALDR) to remove incomplete sequences. Filtered sequences were annotated with SONAR v4.2.
Cell Ranger (10x) was used to analyze reads from hashing libraries.). The resulting count matrix was loaded into Seurat for downstream analysis, and sample identities were assigned using the HTODemux function.

Serum neutralization and ELISA:
Graphpad prism (v.10.1.1) was used to calculate four parameter logistic regressions for reciprocal endpoint neutralization and binding titers of serum antibody.

Epitope binning:
Carterra epitope binning software (v.1.9.2.4505) .

April 2023

Deep mutational scanning:
polyclonal (v6.16)
Full computational analysis pipeline for deep mutational scanning data is at https://github.com/dms-vep/SARS-CoV-2_XBB.1.5_spike_DMS_NIH_Penn_mAbs.

Negative stain EM:
Visualization, docking, molecular analysis, and figure preparation were performed using UCSF ChimeraX. AlphaFold 3 was used to predict the structural models of the Fabs.

Automated pseudovirus neutralization:
For serially diluted samples, the neutralization curve fit was generated on a NAB analysis module on the Labkey web-based server with five-parameter nonlinear regression

Data were analyzed in R version 4.5.1 using custom R scripts that are available upon request.
Nonstandard R packages used to produce figures:
pheatmap (v1.0.13)
circlize (v0.4.17)
dendextend (v1.19.1)

For manuscripts utilizing custom algorithms or software that are central to the research but not yet described in published literature, software must be made available to editors and reviewers. We strongly encourage code deposition in a community repository (e.g. GitHub). See the Nature Portfolio guidelines for submitting code & software for further information.

Data

Policy information about availability of data

All manuscripts must include a data availability statement. This statement should provide the following information, where applicable:

- Accession codes, unique identifiers, or web links for publicly available datasets
- A description of any restrictions on data availability
- For clinical datasets or third party data, please ensure that the statement adheres to our policy

Supplementary Information is available for this paper. Source data is available for this paper. Sequencing data was deposited to the Gene Expression Omnibus (accession code: GSE325987).

Research involving human participants, their data, or biological material

Policy information about studies with human participants or human data. See also policy information about sex, gender (identity/presentation), and sexual orientation and race, ethnicity and racism.

Reporting on sex and gender	We use the term sex in the reporting for this study. Participants were asked to self-report sex. Sex and/or gender were not considered in the design or recruitment of the study, as the study was based on sample availability. The cohort was well balanced between sexes. We did not perform sex-based analysis due to low cohort sizes.
Reporting on race, ethnicity, or other socially relevant groupings	We use the terms race and ethnicity in the reporting for this study. Races identified were White, Asian, and Black. Ethnicity values included Hispanic/Latino or Non-Hispanic/Latino. Participants were asked to self-report race and ethnicity. Race and ethnicity were not considered in the design or recruitment of the study, as the study was based on sample availability. As our cohort is reflective of the University of Pennsylvania research community demographics, it was skewed towards White participants.
Population characteristics	Cohort Demographics are reported in Supplementary Table 5.<br>Type-specific responders after XBB.1.5 vaccination:<br>N=5; Age: age min = 25, age max = 31. age mean = 27.4; Sex: M = 2, F = 3; Race: White = 4, Black =1, Asian = 0<br><br>KP.2 Vaccination:<br>N=14; Age: age min = 25, age max = 71, age mean = 39.9; Sex: M = 6, F = 8; Race: White = 11, Black = 1, Asian = 2; Ethnicity: Non-Hispanic/Latino = 1.
Recruitment	Study participants were recruited by word of mouth. The cohorts were biased based on the University of Pennsylvania research community demographics.
Ethics oversight	Study protocol was approved by the University of Pennsylvania Institutional Review Board (IRB#851465)

Note that full information on the approval of the study protocol must also be provided in the manuscript.

Field-specific reporting

Please select the one below that is the best fit for your research. If you are not sure, read the appropriate sections before making your selection.

☒ Life sciences ☐ Behavioural & social sciences ☐ Ecological, evolutionary & environmental sciences

For a reference copy of the document with all sections, see nature.com/documents/nr-reporting-summary-flat.pdf

Life sciences study design

All studies must disclose on these points even when the disclosure is negative.

Sample size	No sample-size calculations were performed in this study. Sample sizes are reflective of available samples from cohort donors.
Data exclusions	Antibody competition data from our reference II antibody was excluded. Exclusion rationale is detailed in the methods section. Reference antibody II had poor amine coupling to the Carterra LSA HC30M chip, resulting in inconsistent data quality. Antibodies were successfully binned into discrete groups using the remaining reference antibodies.<br><br>For sequencing data, we discarded any nonproductive rearrangements and cells with multiple heavy or light chains.
Replication	Results were confirmed in duplicate unless otherwise noted. Screening assays were performed in single experiments. For flow cytometry and sorting, replicates were not performed due to limited sample availability.
Randomization	Participants were allocated into groups based on reported vaccination history and all available samples were used in analysis. Thus, randomization was not necessary.
Blinding	Investigators were not blinded during data collection. There were no distinct interventions or treatment groups to be blinded to.

Reporting for specific materials, systems and methods

We require information from authors about some types of materials, experimental systems and methods used in many studies. Here, indicate whether each material, system or method listed is relevant to your study. If you are not sure if a list item applies to your research, read the appropriate section before selecting a response.

Materials & experimental systems

n/a	Involved in the study	
☐	☒	Antibodies
☐	☒	Eukaryotic cell lines
☒	☐	Palaeontology and archaeology
☒	☐	Animals and other organisms
☒	☐	Clinical data
☒	☐	Dual use research of concern
☒	☐	Plants

Methods

n/a	Involved in the study	
☒	☐	ChIP-seq
☐	☒	Flow cytometry
☒	☐	MRI-based neuroimaging

Antibodies

Antibodies used

Antibodies used in this study are reported in Supplementary Table 2.

BUV563 anti-CD3 BD Biosciences Cat#748569
BV750 anti-CD19 Biolegend Cat#302262
BV421 anti-CD19 Biolegend Cat#302233
BUV395 anti-CD27 BD Biosciences Cat#563815
BUV661 anti-CD38 BD Biosciences Cat#612969
APC-H7 anti-CD71 BD Biosciences Cat#563671
AF700 anti-IgG Biolegend Cat#337220
AF700 anti-IgG BD Cat#561296
FITC anti-IgA Miltenyi Cat#130-113-475
FITC anti-CD3 Biolegend Cat#300406
FITC anti-CD14 Biolegend Cat#325604
FITC anti-CD16 Biolegend Cat#360716
BB700 anti-IgD BD Biosciences Cat#566538
BV480 anti-IgD BD Biosciences Cat#566138
PE/Dazzle 594 anti-CD21 Biolegend Cat#354922
BV510 anti-IgM BD Biosciences Cat#563113
RB545 anti-CD20 BD Biosciences Cat#569757
PE anti-IgA Miltenyi Cat#130-116-878
TotalSeq™-C0251 anti-human Hashtag 1 Antibody Biolegend Cat#394661
TotalSeq™-C0252 anti-human Hashtag 2 Antibody Biolegend Cat#394663
TotalSeq™-C0253 anti-human Hashtag 3 Antibody Biolegend Cat#394665
TotalSeq™-C0254 anti-human Hashtag 4 Antibody Biolegend Cat#394667
TotalSeq™-C0255 anti-human Hashtag 5 Antibody Biolegend Cat#394669
TotalSeq™-C0256 anti-human Hashtag 6 Antibody Biolegend Cat#394671
TotalSeq™-C0257 anti-human Hashtag 7 Antibody Biolegend Cat#394673
TotalSeq™-C0258 anti-human Hashtag 8 Antibody Biolegend Cat#394675
TotalSeq™-C0259 anti-human Hashtag 9 Antibody Biolegend Cat#394677

TotalSeq™-C0260 anti-human Hashtag 10 Antibody Biolegend Cat#394679
TotalSeq™-C0296 anti-human Hashtag 11 Antibody Biolegend Cat#328941
TotalSeq™-C0262 anti-human Hashtag 12 Antibody Biolegend Cat#394683
TotalSeq™-C0263 anti-human Hashtag 13 Antibody Biolegend Cat#394685
TotalSeq™-C0264 anti-human Hashtag 14 Antibody Biolegend Cat#394687
goat anti-human IgG HRP conjugate Jackson Immunoresearch Cat#109-035-006
anti-VSV-G antibody 1E9F9
SULFO-TAG Anti-Human IgG Antibody MSD Cat#D21ADF-3

Validation

As all antibodies are commercially available, manufacturers completed validation. Further information for validation can be found on the manufacturer's websites.

Eukaryotic cell lines

Policy information about cell lines and Sex and Gender in Research

Cell line source(s)

Expi-293 cells (Thermofisher)
TMPRESS2 Vero E6 cells were gifted by Steven Pohlman
293T-human ACE2 cells were obtained from Drs. Michael Farzan and Huihui Mu at UF Scripps Institute

Authentication

Cell lines were not authenticated.

Mycoplasma contamination

Cell lines were not tested for Mycoplasma contamination.

Commonly misidentified lines
(See ICLAC register)

The cell lines used are not in the commonly misidentified cell lines register.

Plants

Seed stocks

Report on the source of all seed stocks or other plant material used. If applicable, state the seed stock centre and catalogue number. If plant specimens were collected from the field, describe the collection location, date and sampling procedures.

Novel plant genotypes

Describe the methods by which all novel plant genotypes were produced. This includes those generated by transgenic approaches, gene editing, chemical/radiation-based mutagenesis and hybridization. For transgenic lines, describe the transformation method, the number of independent lines analyzed and the generation upon which experiments were performed. For gene-edited lines, describe the editor used, the endogenous sequence targeted for editing, the targeting guide RNA sequence (if applicable) and how the editor was applied.

Authentication

Describe any authentication procedures for each seed stock used or novel genotype generated. Describe any experiments used to assess the effect of a mutation and, where applicable, how potential secondary effects (e.g. second site T-DNA insertions, mosiacism, off-target gene editing) were examined.

Flow Cytometry

Plots

Confirm that:

☒ The axis labels state the marker and fluorochrome used (e.g. CD4-FITC).

☒ The axis scales are clearly visible. Include numbers along axes only for bottom left plot of group (a 'group' is an analysis of identical markers).

☒ All plots are contour plots with outliers or pseudocolor plots.

☒ A numerical value for number of cells or percentage (with statistics) is provided.

Methodology

Sample preparation

Venous blood (30–100mL) was collected into sodium heparin tubes following standard phlebotomy procedures. Plasma was separated by centrifugation at 1800xg for 15 minutes, aliquoted, and stored at −80°C. After plasma removal, the remaining blood was diluted to twice the original blood volume with R1 (RPMI + 1% FBS, 2mM L-glutamine, and 100U penicillin/streptomycin). Diluted blood was layered onto 15mL lymphoprep (STEMCELL Technologies) in SEPMATE tubes (STEMCELL Technologies) and centrifuged at 1200xg for 10 minutes. The peripheral blood mononuclear cell (PBMC) layer was collected, washed once with R1, pelleted, and resuspended in ACK lysis buffer (Thermo Fisher). ACK lysis was performed for 5 minutes at RT and quenched with R1. PBMCs were filtered through a 70µm strainer, counted using a Countess automated counter (Thermo Fisher), and cryopreserved in aliquots of 1×10^7 cells in 90% FBS/10% DMSO at −80°C.

On the day of sorting, 10e6 PBMCs were thawed from cryopreservation and washed with R10 media (RPMI + 10% FBS + 5% Pen-Strep) + 0.05% Benzonase (Millipore). Cells were resuspended in 1mL, counted, and washed again in R10 before proceeding with staining protocols.

Instrument

BD FACSymphony A5 sorter

Instrument	Cytek Aurora
Software	FlowJo v10 or OMIQ were used for flow cytometric analysis.
Cell population abundance	Purity of single-sorted B cells was not determined aside from heavy and light chain sequence analysis. Abundance of sorted populations is represented in figures.
Gating strategy	Gating schemes are depicted in the supplementary figures. For sorting, single cells were gated on FSC-H and FSC-A. Dead cells were excluded with viability dye and were removed by negative gating on a single CD3, CD14, CD16 dump channel. Class-switched B cells were gated as CD19+IgD-, and were then gated positively for IgG or IgA expression. XBB.1.5 RBD+ B cells were gated as double positive for two distinct fluorophores. Two different populations of antigen-specific B cells were then sorted based on their reactivity with WA1 RBD on two distinct fluorophores. For B cell phenotyping, single-cells were gated using FSC-H and FSC-A. Dead cells were excluded using Viability dye. B cells were gated as CD19+CD3-. Memory B cells were gated as non-naive (not CD27-IgD+) non-plasmablasts (not CD27hiCD38hi). Activated B cells were gated as CD21-CD71 +. Before gating for antigen specificity, B cells were negatively gated for binding to a decoy probe.

☒ Tick this box to confirm that a figure exemplifying the gating strategy is provided in the Supplementary Information.
